# Benzodiazepine prescribing for children, adolescents, and young adults from 2006 through 2013: A total population register-linkage study

**DOI:** 10.1371/journal.pmed.1002635

**Published:** 2018-08-07

**Authors:** Anna Sidorchuk, Kayoko Isomura, Yasmina Molero, Clara Hellner, Paul Lichtenstein, Zheng Chang, Johan Franck, Lorena Fernández de la Cruz, David Mataix-Cols

**Affiliations:** 1 Centre for Psychiatry Research, Department of Clinical Neuroscience, Karolinska Institutet, Stockholm, Sweden; 2 Stockholm Health Care Services, Stockholm County Council, Stockholm, Sweden; 3 Department of Medical Epidemiology and Biostatistics, Karolinska Institutet, Stockholm, Sweden; 4 Department of Psychiatry, University of Oxford, Warneford Hospital, Oxford, United Kingdom; London School of Hygiene and Tropical Medicine, UNITED KINGDOM

## Abstract

**Background:**

Pharmacoepidemiological studies have long raised concerns on widespread use of benzodiazepines and benzodiazepine-related drugs (BZDs), in particular long-term use, among adults and the elderly. In contrast, evidence pertaining to the rates of BZD use at younger ages is still scarce, and the factors that influence BZD utilisation and shape the different prescribing patterns in youths remain largely unexplored. We examined the prevalence rates, relative changes in rates over time, and prescribing patterns for BZD dispensation in young people aged 0–24 years in Sweden during the period January 1, 2006–December 31, 2013, and explored demographic, clinical, pharmacological, and prescriber-related attributes of BZD prescribing in this group.

**Methods and findings:**

Through the linkage of 3 nationwide Swedish health and administrative registers, we collected data on 17,500 children (0–11 years), 15,039 adolescents (12–17 years), and 85,200 young adults (18–24 years) with at least 1 dispensed prescription for a BZD during 2006–2013, out of 3,726,818 Swedish inhabitants aged 0–24 years. Age-specific annual prevalence rates of BZD dispensations were adjusted for population growth, and relative changes in rates were calculated between 2006 and 2013. We analysed how BZD dispensation varied by sex, psychiatric morbidity and epilepsy, concurrent dispensation of psychotropic medication, type of dispensed BZD, and type of healthcare provider prescribing the BZD. Prescribing patterns were established in relation to duration (3 months, >3 to ≤6 months, or >6 months), dosage (<0.5 defined daily dosage [DDD]/day, ≥0.5 to <1.5 DDD/day, or ≥1.5 DDD/day), and “user category” (“regular users” [≥0.5 to <1.5 DDD/day for ≥1 year], “heavy users” [≥1.5 DDD/day for ≥1 year], or otherwise “occasional users”). Multinomial regression models were fitted to test associations between BZD prescribing patterns and individual characteristics of study participants. Between 2006 and 2013, the prevalence rate of BZD dispensation among individuals aged 0–24 years increased by 22% from 0.81 per 100 inhabitants to 0.99 per 100 inhabitants. This increase was mainly driven by a rise in the rate among young adults (+20%), with more modest increases in children (+3%) and adolescents (+7%). Within each age category, overall dispensation of BZD anxiolytics and clonazepam decreased over time, while dispensation of BZD hypnotics/sedatives, including Z-drugs, showed an increase between 2006 and 2013. Out of 117,739 study participants with dispensed BZD prescriptions, 65% initiated BZD prescriptions outside of psychiatric services (92% of children, 60% of adolescents, 60% of young adults), and 76% were dispensed other psychotropic drugs concurrently with a BZD (46% of children, 80% of adolescents, 81% of young adults). Nearly 30% of the participants were prescribed a BZD for longer than 6 months (18% of children, 31% of adolescents, 31% of young adults). A high dose prescription (≥1.5 DDD/day) and heavy use were detected in 2.6% and 1.7% of the participants, respectively. After controlling for potential confounding by demographic and clinical characteristics, the characteristics age above 11 years at the first BZD dispensation, lifetime psychiatric diagnosis or epilepsy, and concurrent dispensation of other psychotropic drugs were found to be associated with higher odds of being prescribed a BZD for longer than 6 months, high dose prescription, and heavy use. Male sex was associated with a higher likelihood of high dose prescription and heavy use, but not with being prescribed a BZD on a long-term basis (> 6 months). The study limitations included lack of information on actual consumption of the dispensed BZDs and unavailability of data on the indications for BZD prescriptions.

**Conclusions:**

The overall increase in prevalence rates of BZD dispensations during the study period and the unexpectedly high proportion of individuals who were prescribed a BZD on a long-term basis at a young age indicate a lack of congruence with international and national guidelines. These findings highlight the need for close monitoring of prescribing practices, particularly in non-psychiatric settings, in order to build an evidence base for safe and efficient BZD treatment in young persons.

## Author summary

**Why was this study done?:**

- Benzodiazepines and benzodiazepine-related drugs (BZDs) are among the most widely used psychotropic medications in the world and have long raised public health concerns due to the risk for their users of developing dependence and severe adverse effects, in particular among long-term users.
- The prevalence rates and attributes of BZD use have been well described in adults and the elderly.
- In contrast, evidence regarding BZD prescribing for younger populations is scarce and fragmented, and factors influencing BZD utilisation in children, adolescents, and young adults remain largely unexplored.

**What did the researchers do and find?:**

- We linked nationwide Swedish health and administrative registers on 3,726,818 individuals aged 0–24 years living in Sweden between 2006–2013, among whom 17,500 children (0–11 years), 15,039 adolescents (12–17 years), and 85,200 young adults (18–24 years) were dispensed a BZD prescription at least once during that period.
- The prevalence rate of BZD dispensation has increased by 22% between 2006 and 2013, and has increased particularly in young adults.
- For nearly 65% of all study participants with 1 or more dispensed BZD prescriptions, the first prescription was issued outside specialised psychiatric services. Most first prescriptions originated from primary care (41%) and non-psychiatric specialist settings (24%) such as paediatrics and internal medicine.
- Over 75% of study participants were dispensed other psychotropic medication concurrently with a BZD, raising concerns about the potential risk of drug interactions.
- Almost 30% were prescribed a BZD for longer than 6 months, contradicting international and national guidelines that advise against BZD use beyond 2–4 weeks for adults and generally discourage prescribing BZDs for ages below 18 years.

**What do these findings mean?:**

- There is a need for clinicians, particularly those working in non-psychiatric services, to implement strategies to avoid potentially harmful patterns of prescribing BZDs to young people.
- Young individuals who are prescribed BZDs should be closely monitored, in particular, with respect to duration of BZD treatment and concurrent prescription of other psychotropic drugs.

## Introduction

Widespread use of benzodiazepines and benzodiazepine-related drugs (BZDs) has long raised public health concerns, owing to the risks of developing tolerance, dependence, withdrawal syndromes, and severe adverse effects, particularly among long-term users [1–3]. A recently published perspective piece makes parallels between the contemporary epidemic of overprescribing of opioids and that of BZDs, with the former phenomenon being well acknowledged by clinicians and policymakers, while for the latter one a lesser effort is being made to address today’s prescribing practices [4]. Current knowledge on BZD prescribing, incidence and prevalence rates, and patterns of use mainly rely on data on adults and the elderly, for whom BZDs are often prescribed for managing anxiety symptoms and insomnia [1,3]. International and national guidelines recommend using BZDs for no longer than 2–4 weeks since the risk–benefit ratio beyond that period is debatable [5–8]. Evidence pertaining to the corresponding issues in individuals at younger ages is limited, not least due to BZDs not being recommended as a pharmacological option for treatment of any psychiatric disorders in persons below 18 years of age [9–11]. Indeed, the only firmly established indications of BZDs in this age group are the control of different types of seizures and the treatment of status epilepticus [12–15]. Studies on BZD prescribing in children and adolescents consistently report low annual prevalence rates (0.3%–0.5% in North America [16] and 0.2%–0.9% in Europe [17–20]), while the results of time-trend analyses appear to vary between countries. Over the last 2 decades, BZD prescription rates have increased in children and adolescents in various Western countries [16,21,22], while remaining stable or decreasing in others [17,18,21]. Depending on how each study defines the age boundaries for the category of young adults, BZD prescription rates are reported to range between 1% and 5%, and to mainly increase over time [21,23,24]. Noteworthy is that recent European data on new BZD users show a low and decreasing incidence in the age group below 18 years [25]. This, in light of stable or increasing prevalence rates, points towards a risk for chronic BZD use in this population.

Despite being scarce, studies on paediatric BZD use raise a series of concerns, in particular related to inappropriate prescriptions, concurrent use of psychotropic drugs, changes in the characteristics of the prescribers towards a higher involvement of non-psychiatrists and primary care physicians, and long-term use [16–18,21,26–29]. Few studies have investigated the patterns of BZD use among people below 18 years of age; existing studies used definitions of long-term use ranging from over 1 month [30,31] to over 3 months of cumulative BZD prescribing [17], and the proportion of long-term BZD users varied widely, from 3.3%–5.9% [17,31] to 14.7% [30]. A higher likelihood of long-term use is reported for children and adolescents with psychiatric disorders, epilepsy, and BZD prescriptions initiated by psychiatrists, with less clear results on the role of sex [31]. Given the frequent “off-label” BZD prescribing (i.e., outside of approved indication or age category [32]), and that the risk–benefit ratio of BZD use has not yet been fully clarified for children and adolescents [33–35], it is important to establish predictors and attributes of BZD prescribing patterns in these age groups to serve as an evidence base for guiding clinicians in their prescribing practices.

To fill this important gap in the knowledge base, we aimed to explore the annual prevalence rates of BZD dispensations and relative changes in rates over time among individuals aged below 25 years during 2006–2013. Subsequently, we sought evidence on how BZD dispensations in children, adolescents, and young adults vary by individual characteristics (sex, psychiatric morbidity and epilepsy, and concurrent dispensation of psychotropic medication), type of BZD drug, and type of healthcare provider prescribing the BZD. Furthermore, we aimed to describe BZD prescribing patterns with regard to duration of prescription and prescribed dosage and to explore associations between different patterns and characteristics of the patients.

## Methods

### Data sources and register linkage

The study was set in a population-based cohort constructed through the record linkages of 3 Swedish registers with complete national coverage. The Swedish Prescribed Drug Register (PDR) encompasses data on prescribed medications dispensed across all pharmacies in Sweden from July 2005 onwards, registered using Anatomical Therapeutic Chemical Classification System (ATC) codes, along with dosage, dispensed amount, dispensation date, and type of healthcare provider prescribing the BZD [36]. The PDR does not include treatment indication and medications administered in hospitals. The National Patient Register (NPR) comprises data on clinical diagnoses, coded using the International Classification of Diseases (ICD), from inpatient care (1964 onwards) and specialist outpatient services (2001 onwards), with complete (national) coverage since 1987 and 2001, respectively [37]. The register was validated for an array of diagnoses, with a positive predictive value of 85%–95% overall and up to 97% for psychiatric disorders [37–41]. The Total Population Register (TPR) contains demographic characteristics of Swedish inhabitants from 1968 onwards [42]. The PDR and the NPR are held by the Swedish National Board of Health and Welfare, and the TPR is maintained by Statistics Sweden. The linkage was performed through the unique personal identification number assigned to all Swedish citizens and residents [43]. The study was approved by the Regional Ethical Review Board (2013/5:8) in Stockholm, Sweden. The requirement for informed consent was waived because the study was register-based and the included individuals were not identifiable at any time.

### Study participants

The study participants comprised all individuals aged 0–24 years with at least 1 dispensed BZD prescription between January 1, 2006, and December 31, 2013, according to the PDR. Sex and birth year were collected from the TPR and linked to the data from the PDR. The birth year was used to calculate age at the first and each consecutive BZD dispensation. The participants were categorised into children (0–11 years), adolescents (12–17 years), and young adults (18–24 years). For the analyses over the whole period 2006–2013, categorisation was based on age at first BZD dispensation. For assessing BZD dispensations within each year (e.g., annual prevalence), the actual age of participants in the specific year was used.

### Measures

#### BZD medications and prescribing patterns

BZD medications were defined by ATC codes for benzodiazepine derivatives in anxiolytics (N05BA), hypnotics/sedatives (N05CD), and antiepileptics (N03AE), and for a group of benzodiazepine-related drugs in hypnotics/sedatives known as Z-drugs (N05CF), in the PDR.

For each BZD prescription, the date of dispensation and the dispensed amount (with the defined daily dosage [DDD] as the measurement unit [44]) were also retrieved from the PDR. BZD prescriptions that were dispensed in July–December 2005 were not extracted, to obtain comparable, full-year data for estimating the annual prevalence rates of BZD dispensations in 2006–2013. An “individual treatment period” and an “average daily dosage” of BZD for each treatment period were defined for every participant. A treatment period was defined as a sequence of BZD dispensations where the gap between 2 consecutive dispensations (i.e., between the actual dispensation dates) did not exceed 6 months. The duration of the period was estimated as the length of time between the first and the final BZD dispensation. As the Ordinance on Pharmaceutical Benefits adopted by the Swedish Government allows a maximum 3-month medication supply per prescription [45], the duration of each individual period was then extended by 91 days to ensure capturing the full length of BZD treatment. If there was a gap extending beyond 6 months, the next dispensation was considered as the initiation of a new treatment period. Thus, by definition, each participant could have multiple individual treatment periods of different durations (see S1 Fig for details). Because all drug prescriptions in Sweden are for 3 months as standard, the minimal length of a single treatment period was set at 3 months. Furthermore, an average daily dosage of BZD for each individual treatment period was calculated by dividing the total dispensed amount of BZD in a corresponding treatment period (i.e., the cumulative DDDs dispensed within the corresponding period) by the length of that period (see S1 Fig for details).

Three variables for BZD prescribing patterns were constructed. Duration of prescription was based on the length of an individual treatment period and was subdivided as 3 months (reference category), >3 to ≤6 months, or >6 months. Prescribed dosage was based on an average daily dosage of BZD calculated for the treatment period and was categorised into <0.5 DDD/day (reference category), ≥0.5 to <1.5 DDD/day, or ≥1.5 DDD/day. “User category” was constructed to reflect the benchmarks introduced by the Swedish National Board of Health and Welfare for evaluating quality and efficiency of Swedish healthcare [46]. The variable combined information on the average daily dosage and duration of treatment period, and categorised the individuals as “regular users” (≥0.5 to <1.5 DDD/day for ≥1 year), “heavy users” (≥1.5 DDD/day for ≥1 year), or, otherwise, “occasional users” (reference category). If multiple treatment periods were identified, the highest category of each prescribing pattern was used for analysis (see S1 Fig for details).

#### Concurrent psychotropic medication, psychiatric diagnoses and epilepsy, and type of healthcare provider prescribing the BZD

Data on concurrent psychotropic medications, if they were dispensed up to 6 months prior to or after the dispensation date of a BZD prescription, were retrieved from the PDR from July 1, 2005, to June 30, 2014, to cover a 6-month period for BZD prescriptions that were dispensed at the beginning of 2006 or the end of 2013. Concurrent psychotropic drugs included antipsychotics, antidepressants, mood stabilisers, psychostimulants, drugs used in addictive disorders, analgesics, opioids, non-BZD antiepileptics, non-BZD anxiolytics, and non-BZD hypnotics/sedatives (see S1 Table).

The ICD-10 codes, introduced in 1997, and dates of diagnoses for psychiatric disorders and epilepsy (see S2 Table) were retrieved from the NPR for the period between 1997 and 2013. Two variables were constructed, separately for psychiatric disorders and epilepsy: diagnosed 6 months prior to or after BZD dispensation (for those who collected a BZD between January 1, 2006, and June 30, 2013, to allow a 6-month time window for diagnoses after dispensation) and lifetime diagnosis (if diagnosed ever between 1997–2013).

Data on the healthcare provider category (primary care, psychiatric service, or specialised care other than psychiatry) were retrieved from the PDR for the prescriber who issued the first BZD prescription for each individual.

### Statistical analyses

The study was performed in correspondence with a prespecified analysis plan (see S1 Text). Annual prevalence of BZD dispensations was calculated separately for children, adolescents, and young adults as the proportion of individuals who were dispensed a BZD at least once during the year, for the years 2006 to 2013, out of the total number of inhabitants of the same age category in Sweden in the corresponding year (as reported by Statistics Sweden [47]). Individuals with multiple BZD dispensations within the same year were counted only once. The rates are reported per 100 inhabitants to be interpreted as age-specific annual prevalence of BZD dispensation in the general population of Sweden in 2006–2013.

Subsequently, the attributes of BZD dispensations (i.e., sex, concurrent dispensation of psychotropic drugs, psychiatric diagnoses and epilepsy, type of BZD drug, and healthcare provider category) were analysed among all individuals (0–24 years) with dispensed BZD prescriptions and stratified by age categories. The results are reported in percentages to be interpreted as the proportion with a certain attribute per 100 study participants. The analyses were performed within each year (the denominator included individuals with at least 1 dispensed BZD prescription in a given year) and across the study period (the denominator included all individuals with at least 1 dispensed BZD prescription between 2006 and 2013).

To study changes over time in annual prevalence rates of BZD dispensations in Swedish youths or in proportions of various attributes of BZD dispensations among the study participants, the relative change in measures in 2013 from those in 2006 (the referent value) was calculated: The value estimated in 2006 was subtracted from that in 2013, and the result was divided by the value in 2006. The relative change is reported as a percentage, with positive quantities corresponding to increases in values over time, and negative ones corresponding to decreases. Multinomial logistic regression models were fitted to obtain odds ratios and corresponding 95% confidence intervals for associations of the prescribing patterns with age, sex, lifetime psychiatric diagnoses and epilepsy, and concurrent dispensation of psychotropic drugs. In the modelling strategy, variables were included in the multivariate analysis if they were found significant in the univariate models or if they fulfilled the criteria for being confounders [48].

A series of sensitivity analyses were conducted by repeating the main analyses in a sub-population restricted to individuals without a lifetime diagnosis of epilepsy to capture the BZD prescribing for indications other than seizures. In reporting, we followed the STROBE guidelines for cohort studies (see S1 Checklist). All statistical analyses were performed using SAS, version 9.4 [49].

## Results

### Annual prevalence and relative changes over time in rates of BZD dispensation

**Fig 1:**
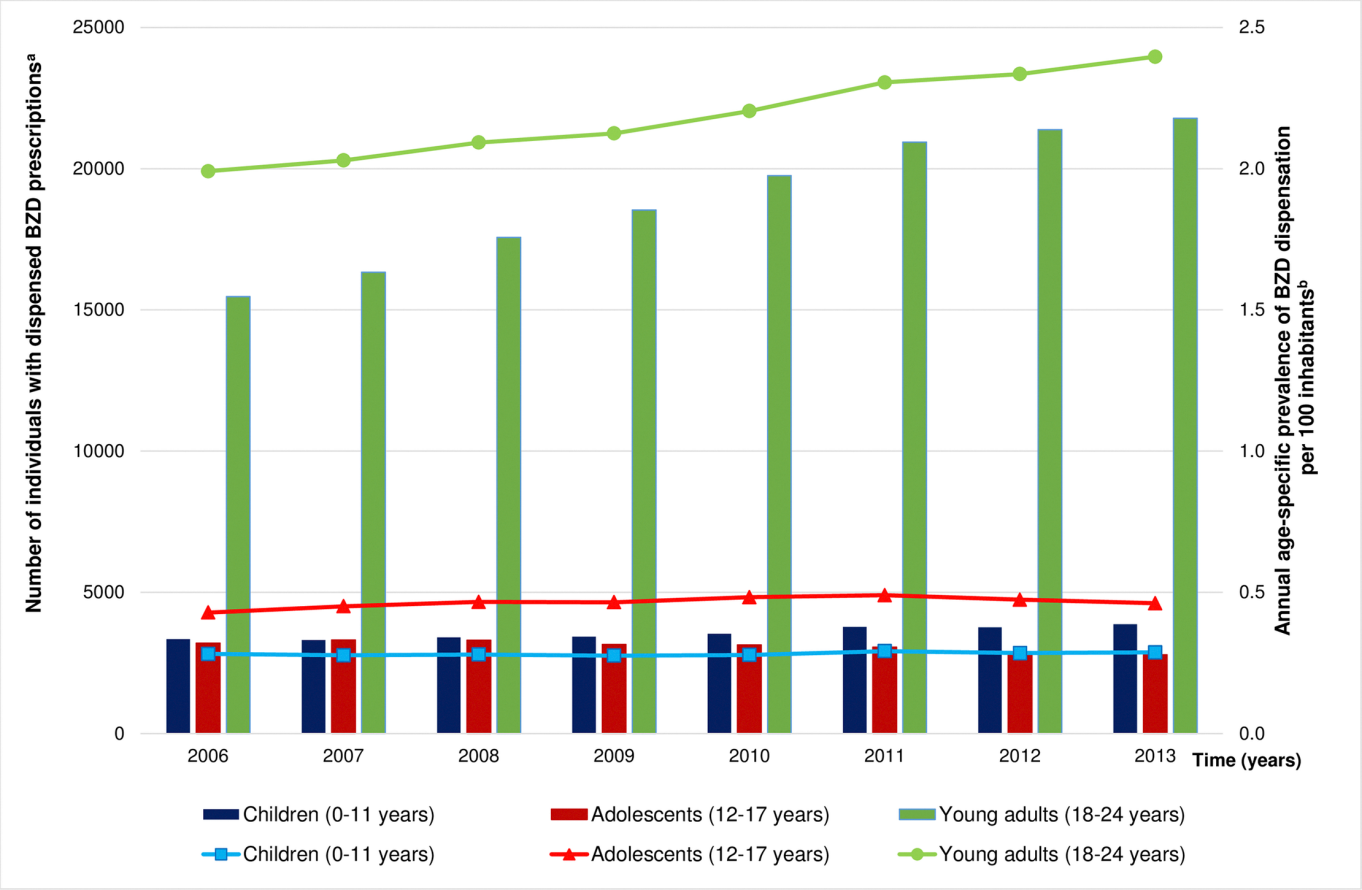
Individuals with dispensed BZD prescriptions by year and the annual age-specific prevalence of dispensations. Relative change in prevalence of BZD dispensation in 2013 from that in 2006 (the value estimated in 2006 is subtracted from that in 2013, and the result is divided by the value in 2006; reported as a percentage, with positive quantities corresponding to increases in values over time): in children +3.5%, in adolescents +6.9%, and in young adults +20.1%. ^a^Bars denote the absolute number of individuals who were dispensed at least 1 BZD prescription in a given year. ^b^Lines denote the annual age-specific prevalence rate of BZD dispensation among the Swedish inhabitants (prevalence rates are adjusted for population growth in the given age groups based on data from Statistics Sweden). BZD, benzodiazepine or benzodiazepine-related drug.

Out of 3,726,818 individuals aged 0–24 years living in Sweden during 2006–2013 (as reported by Statistics Sweden [47]), 117,739 (3.16%) collected at least 1 BZD prescription before their 25th birthday, with prevalence rates ranging from 0.81 per 100 inhabitants in 2006 to 0.99 per 100 inhabitants in 2013, corresponding to a relative increase of 22.2%. Among the study participants, at the time of the first BZD dispensation, 14.8% were children (aged 0–11 years, *n =* 17,500), 12.8% were adolescents (aged 12–17 years, *n =* 15,039), and 72.4% were young adults (aged 18–24 years, *n =* 85,200). The total number of individuals collecting BZDs increased steadily from 2006 to 2013, with sizeable difference between age groups (Fig 1). Throughout the study, the annual prevalence of BZD dispensations in young adults was 5- to 8-fold higher than that in children and adolescents, with the relative change in rate indicating a 20.1% increase from 2006 (prevalence rate of 1.99 per 100 inhabitants) to 2013 (2.39 per 100 inhabitants) for the young adult group. Prevalence rates remained considerably lower and more stable in children (in 2006: 0.28 per 100 inhabitants; in 2013: 0.29 per 100 inhabitants) and adolescents (in 2006: 0.43 per 100 inhabitants; in 2013: 0.46 per 100 inhabitants), with a relative increase of 3.5% and 6.9%, respectively.

### Characteristics of the study sample

Table 1 presents the distribution of demographic and clinical characteristics of the study sample—individuals aged 0–24 years with at least 1 dispensed BZD prescription between 2006 and 2013—for the total sample and within age categories. Among the individuals with dispensed BZD prescriptions, the proportion of females was higher than that of males overall and among adolescents and young adults, but lower in children. Three out of 4 individuals from the total sample collected at least 1 additional class of psychotropic drugs within 6 months of the dispensation of a BZD, and 24.7% of the study participants received 3 or more classes of concurrent medications. The proportions varied across ages, with less than 50% of children and over 80% of adolescents and young adults collecting other psychotropic drugs concomitantly with a BZD. Antidepressants, non-BZD anxiolytics, and non-BZD hypnotics/sedatives prevailed among concurrent drugs in adolescents and young adults, while in children mood stabilisers were collected most frequently.

**Table 1:** Characteristics of children (0–11 years), adolescents (12–17 years), and young adults (18–24 years) with at least 1 dispensed BZD prescription in 2006–2013.

Characteristic of study sample	Age at first BZD dispensation (years)			
	0–24	0–11	12–17	18–24
**Total *n***	117,739	17,500	15,039	85,200
**Sex (%)**				
Males	42.83	55.81	43.37	40.07
Females	57.17	44.19	56.63	59.93
**Concurrent medication (%)**				
*Number of classes of concurrent medication*				
No additional class	24.07	53.55	19.52	18.82
One additional class	27.92	26.39	27.68	28.27
Two additional classes	23.32	11.67	23.67	25.65
Three or more additional classes	24.70	8.39	29.13	27.26
*Class of concurrent medication*^*a*^				
Any antidepressant	49.58	1.38	42.42	60.75
Any psychostimulant	7.43	3.66	13.09	7.21
Any mood stabiliser	17.70	37.20	30.89	11.37
Any antiepileptic (non-BZD)	9.90	15.86	13.52	8.04
Any antipsychotic	14.68	1.95	18.25	16.66
Any anxiolytic/hypnotic/sedative (non-BZD)	38.76	7.79	41.92	44.57
Any analgesic	15.35	9.09	15.91	16.54
Any opioid	15.35	2.43	13.35	18.35
Any drug used in addictive disorders	2.50	0.09	1.78	3.13

^a^Not mutually exclusive.

BZD, benzodiazepine or benzodiazepine-related drug.

Among individuals who collected a BZD up to June 30, 2013 (*n =* 111,182; 94.4% of the study sample), 45.3% had a record of a psychiatric disorder diagnosed within 6 months of BZD dispensation, with anxiety and depression being the most frequent diagnoses (20.3% and 20.1%, respectively). In total, over 11% of study participants were diagnosed with epilepsy within 6-month proximity to BZD dispensation (Table 2). The results for adolescents and young adults corroborated the ones seen in the total sample, while in children only 16.6% had a psychiatric diagnosis, with disruptive behavioural disorders being the most frequent diagnoses (11.8%). The proportion of individuals diagnosed with epilepsy close to BZD dispensation was highest in children (39.7%), followed by that in adolescents (22.7%) and young adults (3.1%). In addition, we assessed lifetime diagnosis of psychiatric disorders and epilepsy using data for all 117,739 study participants. As anticipated, the proportions of participants with lifetime diagnosis were higher than those with diagnosis within 6 months of BZD dispensation, although the most common diagnoses remained unchanged in all age groups.

**Table 2:** Psychiatric disorders and epilepsy diagnosed within 6 months of BZD dispensation (*n =* 111,182) and lifetime diagnosis (*n =* 117,739) in children (0–11 years), adolescents (12–17 years), and young adults (18–24 years) with at least 1 dispensed BZD prescription in 2006–2013.

Disorder	Diagnosis within 6 months of BZD dispensation^a^				Lifetime diagnosis^b^			
	Age at first BZD dispensation (years)				Age at first BZD dispensation (years)			
	0–24	0–11	12–17	18–24	0–24	0–11	12–17	18–24
**Total *n***	111,182	16,621	14,401	80,160	117,739	17,500	15,039	85,200
**Any psychiatric diagnosis (%)**^**c**^	45.31	16.63	52.79	49.91	58.16	22.62	64.99	64.25
Substance use disorders	9.53	0.08	8.15	11.74	16.85	0.33	13.52	20.83
Schizophrenia, schizotypal, and delusional disorders	3.32	0.10	3.34	3.98	4.63	0.15	4.20	5.63
Bipolar disorders	4.27	0.11	5.38	4.94	6.11	0.15	6.60	7.24
Depressive disorders	20.09	0.22	22.93	23.71	29.40	0.49	29.70	35.29
Anxiety disorders	20.29	0.67	20.60	24.30	29.56	1.32	28.05	35.62
Obsessive-compulsive disorder	2.26	0.17	2.94	2.57	3.77	0.28	4.30	4.40
Reaction to severe stress and adjustment disorders	8.30	0.15	9.10	9.84	13.89	0.36	13.23	16.78
Dissociative, somatoform, and other neurotic disorders	1.20	0.14	2.29	1.23	2.73	0.35	3.75	3.05
Mental retardation	3.05	0.04	2.07	3.84	4.76	0.05	2.80	6.07
Autism spectrum disorders	0.18	0.00	0.14	0.23	0.34	0.00	0.19	0.43
ADHD/ADD	3.69	0.00	3.35	4.51	5.04	0.01	4.14	6.23
Disruptive behaviour disorders	4.33	11.76	9.64	1.84	6.28	14.69	12.72	3.42
Emotionally unstable personality disorder	4.90	6.02	10.36	3.68	7.33	8.58	13.87	5.92
Dissocial personality disorder	7.64	3.63	12.23	7.64	12.39	6.33	18.09	12.62
Other personality disorders	0.47	0.30	1.87	0.25	1.44	0.66	3.50	1.23
Nonorganic sleep disorders and insomnias	2.54	0.45	6.31	2.29	4.36	1.01	8.54	4.31
**Epilepsy (%)**	11.10	39.66	22.74	3.08	12.90	42.98	25.96	4.42

Data retrieved from the National Patient Register (NPR), which comprises clinical diagnoses, coded using the International Classification of Diseases, from inpatient care (1964 onwards) and specialist outpatient services (2001 onwards), with complete (national) coverage since 1987 and 2001, respectively. The NPR does not include data on diagnoses recorded in primary care [37].

^a^Data on disorders extracted for study participants who were dispensed a BZD from January 1, 2006, to June 30, 2013, to allow a 6-month time window for diagnoses after dispensation.

^b^Data on disorders extracted for all study participants diagnosed ever between 1997 and 2013.

^c^Not mutually exclusive.

ADD, attention deficit disorder without hyperactivity; ADHD, attention deficit hyperactivity disorder; BZD, benzodiazepine or benzodiazepine-related drug.

### Dispensation of BZD drugs and percentage changes over time

As presented in Table 3, throughout the study, BZD anxiolytics were collected by 57.8% of all study participants dispensed a BZD, with marginal change between 2006 and 2013 (relative decrease of 3.1%). Diazepam and oxazepam were the top BZD anxiolytics dispensed (32.5% and 25.5%, respectively), showing reverse relative changes over time (relative decrease of 17.8% and relative increase of 41.3%, respectively). A similar proportion of study participants collected BZD hypnotics/sedatives, including Z-drugs (60.9%; relative increase of 9.8%), with zopiclone and zolpidem being dispensed most frequently (39.3% and 27.5%, respectively; relative increase of 36.4% and relative decrease of 24.5%, respectively). Over the study period, less than 3% of study participants were dispensed clonazepam (relative decrease of 18.2%).

**Table 3:** Type of BZD medication dispensed to children (0–11 years), adolescents (12–17 years), and young adults (18–24 years) in 2006–2013.

BZD substance	Age at first BZD dispensation (years)							
	0–24 (*n* = 117,739)		0–11 (*n* = 17,500)		12–17 (*n* = 15,039)		18–24 (*n* = 85,200)	
	Percent	Relative change from 2006 to 2013^a^	Percent	Relative change from 2006 to 2013^a^	Percent	Relative change from 2006 to 2013^a^	Percent	Relative change from 2006 to 2013^a^
**Any anxiolytic**	57.77	−3.09	97.39	−5.78	59.10	−12.18	49.39	−2.43
Diazepam	32.50	−17.79	96.52	−8.65	42.68	−30.77	17.56	−32.84
Oxazepam	25.52	+41.28	0.69	+13.06	18.24	+126.01	31.90	+40.72
Potassium clorazepate	<0.01	—^b^	<0.01	—^b^	0.00	—^c^	0.00	—^c^
Lorazepam	0.80	+21.79	0.41	+46.24	1.71	+90.15	0.72	−1.73
Clobazam	0.86	+21.51	3.79	+89.26	1.62	−30.31	0.12	−90.77
Alprazolam	5.96	−34.31	0.06	+3.23	4.57	+62.09	7.42	−38.01
**Any hypnotic or sedative**^**d**^	60.90	+9.84	8.39	+419.12	60.51	+48.50	71.75	+3.00
Nitrazepam	2.50	−19.95	1.99	+39.41	3.07	+6.71	2.50	−40.17
Flunitrazepam	0.87	−45.42	0.09	372.81	0.88	+52.25	1.03	−56.03
Triazolam	0.30	+12.39	0.05	−31.18	0.45	+70.28	0.32	+5.29
Midazolam	1.75	+2,219.60	6.25	+3,258.45	5.71	+1,666.94	0.12	+166.16
Zopiclone	39.29	+36.43	0.47	+16.47	37.85	+56.51	47.52	+38.76
Zolpidem	27.46	−24.47	0.40	+7.05	26.37	+15.37	33.20	−26.60
Zaleplon	1.78	−72.41	0.00	—^c^	1.61	−36.87	2.17	−74.12
**Any antiepileptic**								
Clonazepam	2.80	−18.23	6.66	−1.68	3.85	−16.07	1.82	−47.25

^a^Relative change in proportion of individuals who were dispensed a BZD substance in 2013 from that in 2006 (the value estimated in 2006 is subtracted from that in 2013, and the result is divided by the value in 2006). Relative change is reported as a percentage, with positive quantities corresponding to increases in values over time, and negative ones corresponding to decreases.

^b^Relative change was not calculated as no individuals were dispensed prescriptions for potassium clorazepate in 2006 or in 2013 (1 individual aged 0–11 years was dispensed potassium clorazepate in 2008).

^c^Relative change was not calculated as no individuals in this age category were dispensed a prescription for the BZD substance in question ever during 2006–2013.

^d^Includes benzodiazepine derivatives in hypnotics/sedatives and a group of benzodiazepine-related drugs in hypnotics/sedatives also known as Z-drugs.

BZD, benzodiazepine or benzodiazepine-related drug.

The abovementioned results differed between age groups (Table 3). Children were mainly dispensed BZD anxiolytics (97.4%), but uncommonly dispensed BZD hypnotics/sedatives, including Z-drugs (8.4%), and clonazepam (6.7%). Over the whole study period, diazepam was the dominant BZD drug dispensed to children (95.5%). Adolescents prevailed over young adults in collecting BZD anxiolytics (59.1% versus 49.4%) and clonazepam (3.8% versus 1.8%), while BZD hypnotics/sedatives and Z-drugs were collected at higher rates among young adults compared to among adolescents (71.7% versus 60.5%). Notwithstanding these differences, within each age category overall dispensation of BZD anxiolytics and clonazepam decreased over time, while dispensation of BZD hypnotics/sedatives, including Z-drugs, showed an increase between 2006 and 2013.

### Healthcare provider categories

**Fig 2:**
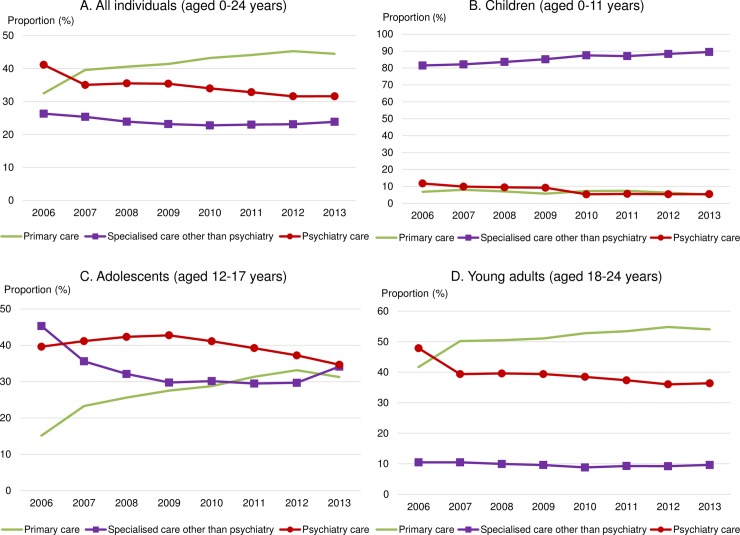
Proportions of the first benzodiazepine or benzodiazepine-related drug (BZD) prescriptions issued within different healthcare provider categories in 2006–2013. Healthcare provider services where the first BZD prescription was issued for (A) all individuals (aged 0–24 years), (B) children (aged 0–11 years), (C) adolescents (aged 12–17 years), and (D) young adults (aged 18–24 years). Shown is the proportion of individuals within each age category who were issued the first BZD prescription in a given healthcare provider service during 2006–2013. Relative changes in proportions of individuals who were issued the first BZD prescription in a given healthcare provider service in 2013 from that in 2006 among all individuals: primary care +36.94%, specialised care other than psychiatry −9.39%, psychiatric care −23.17%; among children: primary care −24.64%, specialised care other than psychiatry +9.79%, psychiatric care −53.80%; among adolescents: primary care +106.68%, specialised care other than psychiatry −24.65%, psychiatric care −12.53%; and among young adults: primary care +29.62%, specialised care other than psychiatry −8.17%, psychiatric care −24.01%.

In the total sample of study participants dispensed a BZD, the first BZD prescription was most commonly issued within non-psychiatric healthcare services (i.e., in primary care, 40.7%, or specialised care other than psychiatry, 24.1%) (Table 4). The healthcare provider category in which BZD prescriptions were initiated differed between age categories, although services outside of psychiatry prevailed across all ages. Only 8.1% of the first BZD prescriptions for children and 40% of such prescriptions for adolescents and young adults were issued at a psychiatric service. Regardless of the age category, the share of BZD prescriptions initiated in psychiatric healthcare services appeared to decrease between 2006 and 2013 (Fig 2).

**Table 4:** Healthcare provider category for where the first BZD prescription was issued for children (0–11 years), adolescents (12–17 years), and young adults (18–24 years) in 2006–2013.

Healthcare provider category (%)	Age at first BZD dispensation (years)			
	0–24 (*n* = 117,739)	0–11 (*n* = 17,500)	12–17 (*n* = 15,039)	18–24 (*n* = 85,200)
Primary care	40.73	6.70	25.41	50.42
Specialised care other than psychiatry^a^	24.13	85.24	34.60	9.72
Psychiatric care	35.15	8.06	39.98	39.86

^a^BZD prescriptions were most frequently initiated by paediatricians for children and adolescents and by internists for young adults.

BZD, benzodiazepine or benzodiazepine-related drug.

### BZD prescribing patterns: Duration of prescription, prescribed dosage, and user category

Throughout the study, 15.4% of the study participants dispensed a BZD were prescribed a BZD for a period of >3 to ≤6 months, while 29.3% of the participants were prescribed a BZD for longer than 6 months (Table 5). The results of univariate multinomial logistic regression indicated that age above 11 years at the first BZD dispensation, a lifetime diagnosis of any psychiatric disorder, and concurrent dispensation of psychotropic drugs were associated with a higher likelihood of being prescribed a BZD for >3 to ≤6 months and for >6 months, while lifetime history of epilepsy was associated with lower odds of having a duration of prescription of >3 to ≤6 months, but with higher odds of being prescribed a BZD for longer than 6 months. When the potential confounding effects of demographic and clinical covariates was accounted for, the observed associations mostly decreased in strength, but all remained significant. Furthermore, in the multivariate model, male sex was associated with a higher likelihood of being prescribed a BZD for >3 to ≤6 months, but no association appeared for a duration of prescription of longer than 6 months.

**Table 5:** BZD prescribing patterns by duration of prescription in 117,739 study participants during the study period (2006–2013).

Covariate	Total *n*^a^	Duration of prescription						
		3 months (reference)	>3 months to ≤6 months			>6 months		
		*n* (%)	*n* (%)	Crude OR (95% CI)	Adjusted^b^ OR (95% CI)	*n* (%)	Crude OR (95% CI)	Adjusted^b^ OR (95% CI)
**Whole cohort**	117,739	65,149 (55.33)	18,093 (15.37)			34,497 (29.30)		
**Sex**								
Females	67,313	36,758 (54.61)	10,357 (15.39)	1.00	1.00	20,198 (30.01)	1.00	1.00
Males	50,426	28,391 (56.30)	7,736 (15.34)	0.98 (0.94–1.00)	1.09 (1.05–1.13)	14,299 (28.36)	0.92 (0.89–0.94)	1.00 (0.97–1.03)
**Age at first BZD dispensation**								
0–11 years	17,500	13,342 (76.24)	1,075 (6.14)	1.00	1.00	3,083 (17.62)	1.00	1.00
12–17 years	15,039	8,137 (54.11)	2,192 (14.58)	3.34 (3.09–3.61)	1.83 (1.68–1.99)	4,710 (31.32)	2.50 (2.37–2.64)	1.24 (1.16–1.32)
18–24 years	85,200	43,670 (51.26)	14,826 (17.40)	4.21 (3.95–4.50)	2.27 (2.10–2.45)	26,704 (31.34)	2.64 (2.53–2.76)	1.49 (1.41–1.58)
**Any lifetime psychiatric diagnosis**^**c**^	68,476	28,244 (41.25)	12,265 (17.91)	2.75 (2.66–2.85)	1.75 (1.68–1.82)	27,967 (40.84)	5.60 (5.42–5.77)	3.52 (3.41–3.64)
**Lifetime diagnosis of epilepsy**^**d**^	15,191	7,829 (51.54)	1,570 (10.34)	0.70 (0.66–0.74)	0.87 (0.81–0.93)	5,792 (38.13)	1.48 (1.42–1.53)	1.66 (1.58–1.75)
**Concurrent dispensation of any psychotropic medication**^**e**^	89,400	40,514 (45.32)	15,938 (17.83)	4.50 (4.29–4.72)	3.18 (3.02–3.35)	32,948 (36.85)	12.93 (12.26–13.64)	7.04 (6.66–7.45)

^a^Total number of individuals in each row represents 100%.

^b^Adjusted for all variables in the table.

^c^Reference category is the individuals without any lifetime psychiatric diagnosis.

^d^Reference category is the individuals without lifetime epilepsy.

^e^Reference category is the individuals without any concurrent psychotropic medication, i.e., psychotropic medication dispensed within 6 months prior to or after BZD dispensation.

BZD, benzodiazepine or benzodiazepine-related drug; OR, odds ratio.

In addition, over 13% of the participants were prescribed a BZD with an average daily dosage of ≥0.5 to <1.5 DDD/day, while an average daily dosage of ≥1.5 DDD/day was observed in 2.6% of individuals (see S3 Table). Regardless of the adjustment strategy, both categories of prescribed dosage were associated with male sex, age at first BZD dispensation above 11 years, a lifetime diagnosis of any psychiatric disorder, and concurrent dispensation of psychotropic medication. In the analysis that controlled for the effect of other covariates, lifetime history of epilepsy had a significant association with a higher likelihood of being prescribed a BZD with an average daily dosage of ≥1.5 DDD/day.

Furthermore, 6.1% of study participants fulfilled the criteria for being regular users, while 1.7% were considered heavy users (see S4 Table). In the multivariate model, male sex was associated with higher odds of being a heavy user, but not a regular user. The odds of being a regular or heavy user increased with age at first dispensation, in the presence of lifetime psychiatric disorders or epilepsy, and with concurrent dispensation of other psychotropic drugs.

### Sensitivity analyses: BZD prescribing in sub-population without lifetime diagnosis of epilepsy

Because treatment of seizures is the most common indication for BZDs in early life [9], we restricted the analyses to study participants without lifetime diagnosis of epilepsy (*n =* 102,548). The results were largely unchanged (see S5–S8 Tables). The only exceptions were the analyses of BZD prescribing patterns, where a sizeable reduction in the proportion of individuals in the highest category of each pattern was observed (see S9–S11 Tables). The differences, however, appeared only among children, while the proportions of adolescents and young adults with a BZD being prescribed on a long-term basis (> 6 months), high average daily dosage (≥1.5 DDD/day), and heavy use remained similar to the ones detected by the main analyses.

## Discussion

This total population register-based study is among the first to systematically evaluate BZD dispensation and its attributes among children, adolescents, and young adults at a nationwide level. There were 5 principal findings. First, the study indicated a 22% increase in the prevalence rate of BZD dispensation between 2006 (0.81 per 100 inhabitants) and 2013 (0.99 per 100 inhabitants) in the population aged 0–24 years. This increase was mainly driven by a steady rise in the rate among young adults, with more modest increases in children and adolescents. Second, in all age categories of those with dispensed BZD prescriptions, a high proportion of polypharmacy was observed, with almost half of children and over 80% of adolescents and young adults having been dispensed other psychotropic drugs concomitantly with a BZD. Third, off-label BZD prescription was common. This was particularly notable in adolescents, among whom a substantial proportion were dispensed zopiclone and zolpidem—drugs that are not approved for ages below 18 years, according to international and Swedish pharmaceutical guidelines [5,50]—with a marked increase in dispensations of both drugs between 2006 and 2013 in this age group. Fourth, although the type of healthcare provider that initiated BZD prescriptions varied between the age categories, the prescribers were mainly outside of specialised psychiatric services; approximately 65% of all prescriptions originated either in primary care or non-psychiatric specialist services. Fifth, the most alarming results came from the analyses of prescribing patterns, with an unexpectedly high proportion of individuals across all ages being prescribed a BZD on a long-term basis—nearly every fifth child and every third adolescent and young adult among those who received a BZD in 2006–2013 were prescribed such medication for longer than 6 months. An elevated likelihood of long-term prescribing was associated with age above 11 years at first BZD dispensation, lifetime diagnosis of any psychiatric disorder or epilepsy, and concomitant dispensation of other psychotropic medication. Sensitivity analyses provided additional evidence of long-term prescribing being a common phenomenon, as the proportion of adolescents and young adults who were prescribed a BZD for longer than 6 months remained large even when individuals with lifetime diagnosis of epilepsy were excluded. Among children, although the proportion of those prescribed a BZD on a long-term basis dropped by 80% after excluding those with lifetime diagnosis of epilepsy, BZD prescribing for longer than 6 months remained present in nearly 4% of study participants in this age group.

A general data scarcity on BZD prescribing in younger populations and differences in methodological approaches between studies makes it difficult to find a direct comparison for the present findings, which integrate a wide spectrum of attributes of BZD dispensation in young people. Nonetheless, the rates of BZD dispensations in children, adolescents, and young adults from our study corroborated the results of cross-national and national projects from Europe and Canada [16,17,21], although they were higher than the rates reported for Norway and Iceland [18,28]. Likewise, our findings of increasing rates over time were supported by cross-European [21] and Canadian data [16], but differed from the decreasing rates seen among children and adolescents in Norway, Ireland, and Denmark [17,18,26]. While interpreting the results on BZD dispensations in young populations, it is important to keep in mind that there is no firmly established indication for BZD treatment in child and adolescent psychiatry [9]—that is also true for Sweden [8,51,52]. In Swedish clinical guidelines for adults (i.e., age 18 years and above), the use of, for example, oxazepam is limited to alcohol withdrawal and delirium tremens and as a second-line drug for temporary management of anxiety symptoms, and zopiclone is a first-line drug for insomnia [8,51,53]. Additionally, guidelines recommend diazepam and midazolam to be used for all ages for treatment of acute epilepsy, while for status epilepticus, midazolam is listed for patients aged below 18 years and diazepam for older individuals [51]. In relation to the abovementioned, our results on concomitant dispensation of psychotropic drugs add to the concerns previously raised about a high prevalence of psychotropic polypharmacy in children and adolescents [17,20,25,27,28,54]. The concerns stem, particularly, from the evidence of enhanced adverse effects of BZDs, including central nervous system depression and respiratory depression, in the presence of other central nervous system depressants (e.g., antipsychotics, antidepressants, and non-BZD anxiolytics) or respiratory depressants (e.g., opioid analgesics) [20]. BZD concurrency with prescription opioids (which in our study appeared in 13% of adolescents and 18% of young adults) gives another cause for concern due to the increased risk of overdose and death among polydrug users [55,56]. Given the restrictions posed by the guidelines on clinical utilisation of BZDs, our findings of dispensed prescriptions for zopiclone and zolpidem in individuals aged below 18 years are undoubtedly worrisome, but not uncommon, and corroborate the results from studies in other countries with smaller sample sizes but comparable age categories [17,34]. In addition, our finding of a large proportion of BZD prescriptions being initiated outside psychiatric services corresponds to similar practices reported for patients aged 0–20 years in North American studies and aged 0–17 years in Icelandic studies, although those studies cover periods only up to 2010 [24,28,57]. The authors of those studies put forward the finding of the increase in visits to non-psychiatrists that appears to coincide with an increase in prescribing of BZDs to young individuals [24,28,57]. The issue was further addressed in a systematic review that demonstrated a substantial diversity in knowledge, attitudes, and awareness about balancing the risks and benefits of BZD treatment among primary care specialists [58]. It is worth mentioning that, over the study period, the number of adolescents and young adults seeking psychiatric care in Sweden increased significantly, with anxiety and depression being the top diagnoses [59,60]. The same age groups also demonstrated a recent increase in receiving psychiatric diagnoses in primary care services and specialised care other than psychiatry [59]. Additionally, there has been a shift in the Swedish healthcare services towards expecting primary care to act as a “first line of psychiatry” [61].

Our finding of an unexpectedly high proportion of individuals who were prescribed BZDs for longer than 6 months deserves special attention. Although there is no consensus between pharmacoepidemiological studies on the definition of long-term use [2], international clinical guidelines strongly advise limiting BZD treatment to the shortest possible period of 2–4 weeks due to side-effects and dependence and withdrawal concerns [1,3,6,7]. A recent systematic review on long-term BZD use reported an average prevalence of 24% (95% CI 13% to 36%) among BZD users of all ages, though the evidence mostly came from studies on adult and elderly users, with the definition of long-term use varying from 1 month to over 12 months [2]. As discussed above, the Swedish guidelines do not recommend BZDs for treatment of psychiatric disorders among individuals aged below 18 years, and the maintenance therapy to prevent seizures in patients with genuine epilepsy or other convulsive disorders cannot explain the observed prescribing patterns. Given that in our study nearly 30% of the participants were prescribed a BZD for longer than 6 months (even after excluding patients with lifetime epilepsy diagnosis, who represented 11% of the study population), further examination is required of potential predictors and mediators of this long-term BZD prescribing pattern. As we assessed only the likelihood of such a pattern, it was not possible to infer causality, and, therefore, no risk or protective factors could be clearly established in this study. However, the results pointed out several attributes of long-term BZD prescribing, including age of onset older than 11 years, psychiatric comorbidities and epilepsy, and concomitant dispensation of other psychotropic medications. These findings are consistent with previous research [2,31,62] and highlight the need for thorough monitoring of BZD prescribing practices for drug–drug interactions, adverse effects, and guideline adherence.

The study has several strengths, including the use of the official Swedish registers with complete national coverage, extensive collection of clinical and demographic data, and timely and routine updating. The robust and comprehensive nature of the present data minimises the potential influence of sampling and reporting error and recall bias. The PDR covers all dispensed drugs regardless of reimbursement status and service provider characteristics, which makes our data representative of the prescribing practices across all healthcare levels in Sweden. In addition, the use of population-based registers ensures generalisability of the results at the national level. Our study has, however, some limitations. First, the PDR does not contain data on the indications for prescriptions, which may have affected our ability to fully analyse a relation between comorbid diagnoses and BZD dispensations among the study participants. In addition, we were unable to retrieve data on disorders diagnosed at primary care services, which could possibly have resulted in an underestimation of the proportion of individuals with comorbidity if they were diagnosed outside of hospitals and specialised care services. Second, we lacked data on the diagnosis of febrile convulsions (ICD-10 code: R56.0) in the NPR. Febrile convulsions are rather common below 6 years of age, and such data, if available, might have given an additional insight into the associations between clinical diagnoses and BZD dispensations, particularly among young children. Third, the data collection was limited to the diagnoses coded with the ICD 10th revision (i.e., starting from 1997). This may have led to missing diagnoses of psychiatric disorders or epilepsy in people born between 1982 and 1997 if these diagnoses were recorded in the NPR before 1997 and were not mentioned again later. Fourth, although the initial analyses included 3 types of BZD prescribing patterns, only the duration of prescription was discussed as a principal finding. It was difficult to draw conclusions about the other 2 patterns as many BZD substances have several indications and, hence, different dosages may be recommended for different conditions. In the absence of information on the exact indication, we wanted to avoid misinterpreting the patterns of prescribed dosage and user category, and, therefore, the results reported on these patterns should be interpreted as suggestive. Fifth, our analyses rest on dispensation data, and, hence, we cannot be certain that the medication was used in proximity to the date of dispensation and by the person it had been prescribed to. However, it is unlikely that the proportion of individuals who collected but did not use BZDs varied across the study period [25]. Lastly, the PDR only covers the period starting July 2005, making it impossible to analyse the rates and patterns of BZD prescriptions dispensed prior to initiation of the register.

Notwithstanding the limitations, to our knowledge, this is the first study to explore BZD prescribing patterns from different perspectives, namely duration and drug dosage, and to identify individual characteristics that are associated with different prescribing patterns in young people. Our study delineates the directions for further research since less consistency still exists in relation to other potential attributes of BZD prescribing, in particular long-term prescribing, including sex, pharmacological features of BZD substances, concurrent prescription of distinct BZD classes, and combination of BZD treatment with certain modalities of psychotherapy. Further work is needed to get insight on the underlying mechanisms and risk and protective factors that shape the patterns of BZD prescription and use among youths. Our findings have clear implications across a range of clinical settings as well as for public policy. The results highlighted a need to devise and implement strategies to avoid potentially harmful patterns of prescribing BZDs to young people. This can only be achieved by involving primary care—the source of the majority of BZD prescriptions—but also other categories of prescribers, including but not limited to specialised care in paediatrics, internal medicine, and psychiatry. Prescribing practices need to be addressed using education, feedback, and peer support. For patients with problematic use, the UK National Institute for Health and Care Excellence guidelines recommend using stepped care models, where patients are given the least intrusive recommended treatment [63]. Furthermore, given the increased availability of addictive drugs via the internet, reductions in harmful prescribing need to be coordinated with availability policies involving, for example, customs and policing.

### Conclusions

The annual prevalence rate of BZD dispensation in the Swedish population aged 0–24 years increased substantially between 2006 and 2013. This increase was mainly driven by a steady rise in the rate of BZD dispensation to young adults, accompanied by an increasing proportion of off-label prescriptions. Polypharmacy was the norm rather than the exception, pointing to the potential risk of drug interactions. Contradicting clinical guidelines, almost 30% of the study participants were prescribed BZDs on a long-term (>6 months) basis. During the study period, growing shares of prescriptions to individuals in all age categories were initiated in primary care or non-psychiatric specialty settings. It is critical to improve prescribing practices through close monitoring of BZD utilisation in young people, strengthening the adherence to BZD prescribing guidelines among primary care practitioners and other non-psychiatric specialists, and improving communication between non-specialised care and specialised psychiatric services.

## Supporting information

S1 Checklist Strengthening the Reporting of Observational Studies in Epidemiology (STROBE) checklist. (DOCX)

S1 Fig Visual representation of individual treatment periods, average daily dosage, and prescribing patterns for a hypothetical cohort member with multiple dispensed prescriptions for benzodiazepines and benzodiazepine-related drugs (BZDs). Prescribing patterns of a hypothetical case (with multiple dispensations A–G): (i) Duration of prescription: Period 2 is considered for the analysis as it is the longest period. Period 2 defines this case’s category of duration of prescription as >6 months. (ii) Prescribed dosage: Period 3 is considered for the analysis as it has the largest average daily dosage. Period 3 defines a this case’s category of prescribed dosage as ≥0.5 to <1.5 defined daily dosage (DDD)/day. (iii) User category: Period 2 is used to define this case as being in the category of regular user, ≥0.5 to <1.5 DDD/day for ≥1 year.
(TIF)

S1 Table Psychotropic medications included in the study and their corresponding ATC codes. (DOCX)

S2 Table ICD-10 diagnostic codes included in the study. (DOCX)

S3 Table BZD prescribing patterns by average daily dosage in 117,739 study participants during the study period (2006–2013). (DOCX)

S4 Table BZD prescribing patterns by user category in 117,739 study participants during the study period (2006–2013). (DOCX)

S5 Table Characteristics of children (0–11 years), adolescents (12–17 years), and young adults (18–24 years) without lifetime diagnosis of epilepsy with at least 1 dispensed BZD prescription in 2006–2013. (DOCX)

S6 Table Psychiatric disorders diagnosed within 6 months of BZD dispensations (*n* = 96,498) and lifetime diagnosis (*n* = 102,548) in children (0–11 years), adolescents (12–17 years), and young adults (18–24 years) without lifetime diagnosis of epilepsy with at least 1 dispensed BZD prescription in 2006–2013. (DOCX)

S7 Table Type of BZD medication dispensed to children (0–11 years), adolescents (12–17 years), and young adults (18–24 years) without lifetime diagnosis of epilepsy in 2006–2013. (DOCX)

S8 Table Healthcare provider category for where the first BZD prescription was issued for children (0–11 years), adolescents (12–17 years), and young adults (18–24 years) without lifetime diagnosis of epilepsy in 2006–2013. (DOCX)

S9 Table BZD prescribing patterns by duration of prescription in 102,548 study participants without lifetime diagnosis of epilepsy during the study period (2006–2013). (DOCX)

S10 Table BZD prescribing patterns by average daily dosage in 102,548 study participants without lifetime diagnosis of epilepsy during the study period (2006–2013). (DOCX)

S11 Table BZD prescribing patterns by user category in 102,548 study participants without lifetime diagnosis of epilepsy during the study period (2006–2013). (DOCX)

S1 Text Study protocol and analysis plan. (DOCX)

## Abbreviations

ATC: Anatomical Therapeutic Chemical Classification System
BZD: benzodiazepine or benzodiazepine-related drug
DDD: defined daily dosage
NPR: National Patient Register
PDR: Prescribed Drug Register
TPR: Total Population Register
