# Supplementary material for: Benzodiazepine prescribing for children, adolescents, and young adults from 2006 through 2013: A total population register-linkage study
Source: PLoS Med. 2018 Aug 7;15(8):e1002635. doi: 10.1371/journal.pmed.1002635 (PMC6080748; doi:10.1371/journal.pmed.1002635)
Supplement: S1 Checklist — (DOCX) [file pmed.1002635.s001.docx]

**S1 Checklist. Strengthening the Reporting of Observational Studies in Epidemiology (STROBE) checklist**

This is a population-based study that applies a register-based cohort design with prevalent outcome measures. In reporting we followed the STROBE guidelines ***for cohort studies***. Below we provide comments on the study procedures and the reporting approaches along with the corresponding page numbers.

|  | Item No | Recommendation | In the manuscript |
| --- | --- | --- | --- |
| **Title and abstract** | 1 | (*a*) Indicate the study’s design with a commonly used term in the title or the abstract | Title (a total population register-linkage study) |
|  |  | (*b*) Provide in the abstract an informative and balanced summary of what was done and what was found | Abstract |
| Introduction | | |  |
| Background/rationale | 2 | Explain the scientific background and rationale for the investigation being reported | Introduction |
| Objectives | 3 | State specific objectives, including any prespecified hypotheses | Last paragraph in Introduction |
| Methods | | |  |
| Study design | 4 | Present key elements of study design early in the paper | “Data sources and register linkage” in Methods |
| Setting | 5 | Describe the setting, locations, and relevant dates, including periods of recruitment, exposure, follow-up, and data collection | “Measures” in Methods |
| Participants | 6 | (*a*) *Cohort study*—Give the eligibility criteria, and the sources and methods of selection of participants. Describe methods of follow-up  *Case-control study*—Give the eligibility criteria, and the sources and methods of case ascertainment and control selection. Give the rationale for the choice of cases and controls  *Cross-sectional study*—Give the eligibility criteria, and the sources and methods of selection of participants | “Study participants” in Methods |
|  |  | (*b*) *Cohort study*—For matched studies, give matching criteria and number of exposed and unexposed  *Case-control study*—For matched studies, give matching criteria and the number of controls per case | Not applicable (N/A) |
| Variables | 7 | Clearly define all outcomes, exposures, predictors, potential confounders, and effect modifiers. Give diagnostic criteria, if applicable | “Measures” in Methods |
| Data sources/ measurement | 8* | For each variable of interest, give sources of data and details of methods of assessment (measurement). Describe comparability of assessment methods if there is more than one group | *“Measures” in Methods*  *(this is a register-based study with uniformed approaches to collect data from the registers on all study participants; the linkage was performed by the means of the unique personal identification number assigned to all Swedish citizens and residents)* |
| Bias | 9 | Describe any efforts to address potential sources of bias | N/A.  The study is based on the registers with complete national coverage (page 8) that minimizes the risk of bias; this issue is addressed in the “Strengths and limitations” |
| Study size | 10 | Explain how the study size was arrived at | “Study participants” and “Statistical analyses” in Methods |
| Quantitative variables | 11 | Explain how quantitative variables were handled in the analyses. If applicable, describe which groupings were chosen and why | “Measures” in Methods |
| Statistical methods | 12 | (*a*) Describe all statistical methods, including those used to control for confounding | “Statistical analyses” in Methods |
|  |  | (*b*) Describe any methods used to examine subgroups and interactions | “Statistical analyses” in Methods |
|  |  | (*c*) Explain how missing data were addressed | N/A (as the registers with complete national coverage had been used) |
|  |  | (*d*) *Cohort study*—If applicable, explain how loss to follow-up was addressed  *Case-control study*—If applicable, explain how matching of cases and controls was addressed  *Cross-sectional study*—If applicable, describe analytical methods taking account of sampling strategy | N/A |
|  |  | (*e*) Describe any sensitivity analyses | Last paragraph in “Statistical analyses” |

Continued on next page

| Results |  |  | Manuscript page number |
| --- | --- | --- | --- |
| Participants | 13* | (a) Report numbers of individuals at each stage of study—eg numbers potentially eligible, examined for eligibility, confirmed eligible, included in the study, completing follow-up, and analysed | “Annual prevalence and time trends of BZD dispensations” in Results |
|  |  | (b) Give reasons for non-participation at each stage | N/A (due to the national coverage of the registers) |
|  |  | (c) Consider use of a flow diagram | N/A |
| Descriptive data | 14* | (a) Give characteristics of study participants (eg demographic, clinical, social) and information on exposures and potential confounders | Table 1-4, Fig 1, Fig 2A-D |
|  |  | (b) Indicate number of participants with missing data for each variable of interest | N/A (due to the completeness of the registers; the registers have reached the complete national coverage prior to the study as described in “Data sources and register linkage”) |
|  |  | (c) *Cohort study*—Summarise follow-up time (eg, average and total amount) | N/A (the study did not apply the statistical approach for which time-to-event variables should be considered; the analyses have been performed on the prevalent cases) |
| Outcome data | 15* | *Cohort study*—Report numbers of outcome events or summary measures over time | “Annual prevalence and time trends of BZD dispensations” and Fig 1 (for the annual age-specific prevalence);  “BZD prescribing patterns: duration of prescription, prescribed dosage, and “user category” and Table 5 (for the proportion of persons with different usage patterns) |
|  |  | *Case-control study—*Report numbers in each exposure category, or summary measures of exposure |  |
|  |  | *Cross-sectional study—*Report numbers of outcome events or summary measures |  |
| Main results | 16 | (*a*) Give unadjusted estimates and, if applicable, confounder-adjusted estimates and their precision (eg, 95% confidence interval). Make clear which confounders were adjusted for and why they were included | Table 5 |
|  |  | (*b*) Report category boundaries when continuous variables were categorized | Age was categorized in three categories as explained in the “Methods” |
|  |  | (*c*) If relevant, consider translating estimates of relative risk into absolute risk for a meaningful time period |  |
| Other analyses | 17 | Report other analyses done—eg analyses of subgroups and interactions, and sensitivity analyses | S5-S11 Tables |
| Discussion | | |  |
| Key results | 18 | Summarise key results with reference to study objectives | First paragraph in Discussion |
| Limitations | 19 | Discuss limitations of the study, taking into account sources of potential bias or imprecision. Discuss both direction and magnitude of any potential bias | Fourth paragraph in Discussion |
| Interpretation | 20 | Give a cautious overall interpretation of results considering objectives, limitations, multiplicity of analyses, results from similar studies, and other relevant evidence | Second and third paragraphs in Discussion |
| Generalisability | 21 | Discuss the generalisability (external validity) of the study results | Fourth paragraph in Discussion |
| Other information | | |  |
| Funding | 22 | Give the source of funding and the role of the funders for the present study and, if applicable, for the original study on which the present article is based | Reported via electronic submission |

*Give information separately for cases and controls in case-control studies and, if applicable, for exposed and unexposed groups in cohort and cross-sectional studies.

**Note:** An Explanation and Elaboration article discusses each checklist item and gives methodological background and published examples of transparent reporting. The STROBE checklist is best used in conjunction with this article (freely available on the Web sites of PLoS Medicine at http://www.plosmedicine.org/, Annals of Internal Medicine at http://www.annals.org/, and Epidemiology at http://www.epidem.com/). Information on the STROBE Initiative is available at www.strobe-statement.org.
