# Supplementary figures and images for: Benzodiazepine prescribing for children, adolescents, and young adults from 2006 through 2013: A total population register-linkage study
Source: PLoS Med. 2018 Aug 7;15(8):e1002635. doi: 10.1371/journal.pmed.1002635 (PMC6080748; doi:10.1371/journal.pmed.1002635)

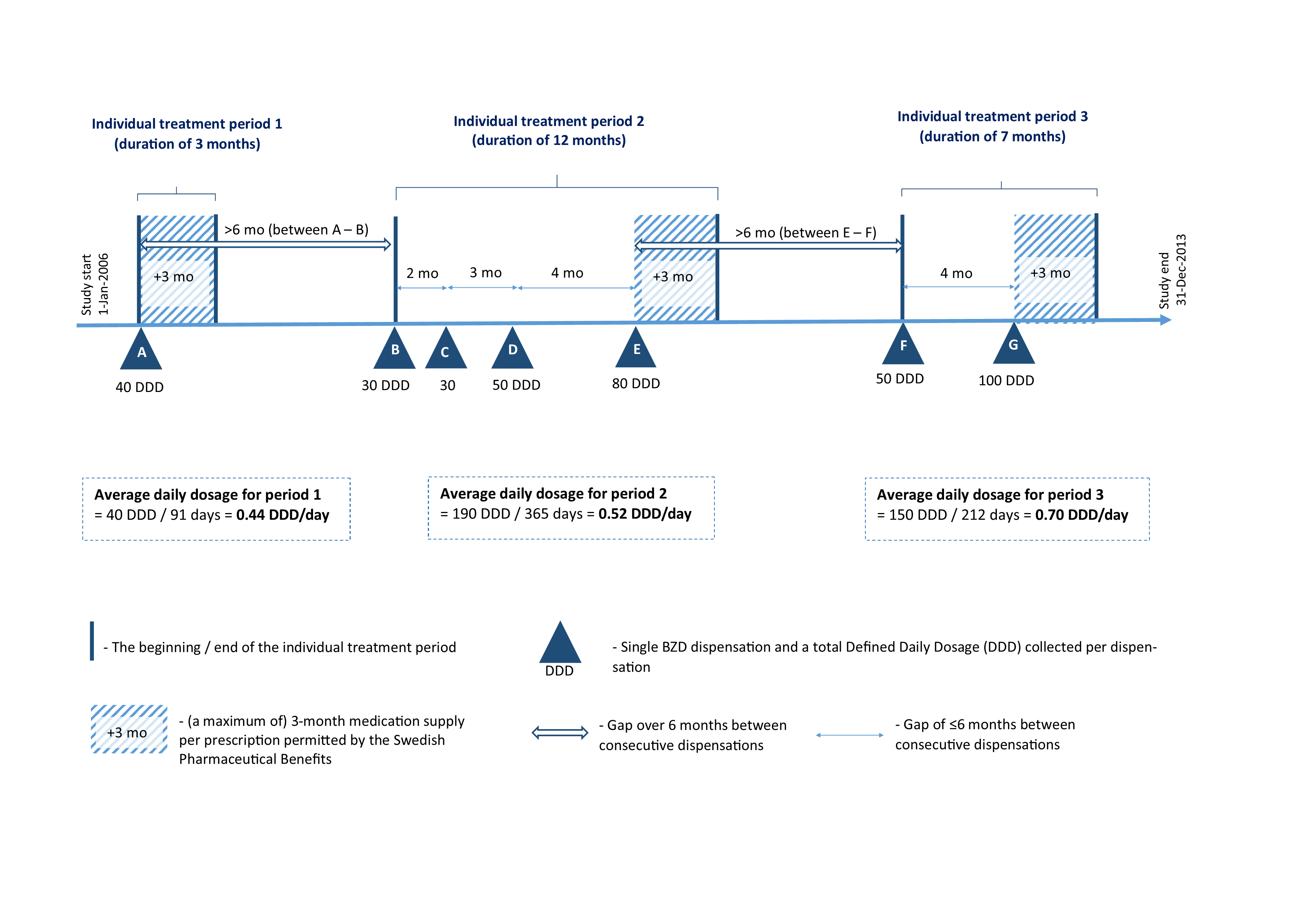

Supplement: S1 Fig — Prescribing patterns of a hypothetical case (with multiple dispensations A–G): (i) Duration of prescription: Period 2 is considered for the analysis as it is the longest period. Period 2 defines this case’s category of duration of prescription as >6 months. (ii) Prescribed dosage: Period 3 is considered for the analysis as it has the largest average daily dosage. Period 3 defines a this case’s category of prescribed dosage as ≥0.5 to <1.5 defined daily dosage (DDD)/day. (iii) User category: Period 2 is used to define this case as being in the category of regular user, ≥0.5 to <1.5 DDD/day for ≥1 year. (TIF) [file pmed.1002635.s002.tif]
