# Supplementary material for: Benzodiazepine prescribing for children, adolescents, and young adults from 2006 through 2013: A total population register-linkage study
Source: PLoS Med. 2018 Aug 7;15(8):e1002635. doi: 10.1371/journal.pmed.1002635 (PMC6080748; doi:10.1371/journal.pmed.1002635)
Supplement: S1 Table — (DOCX) [file pmed.1002635.s003.docx]

**S1 Table. Psychotropic medications included in the study and their corresponding ATC codes.**

| **Study covariate** | **ATC code** |
| --- | --- |
| Antidepressants | N06A |
| Psychostimulants (centrally acting sympathomimetics) | N06BA |
| Mood stabilisers | N03AF01, N03AF02, N03AG01, N03AX09, N03AN01 |
| Antiepileptics (non-BZD) | N03 (except N03AE)^a^ and (except N03AF01, N03AF02, N03AG01, N03AX09, N03AN01)^b^ |
| Antipsychotics | N05A |
| Anxiolytics/hypnotics/sedatives (non-BZD) | N05B (except N05BA)^c^  N05C (except N05CD, N05CF)^d^ |
| Analgesics | N02B, N02C |
| Opioids | N02A |
| Drugs used in addictive disorders | N07B |

^a^Excluded from non-BZD antiepileptics and used to select benzodiazepine derivatives in antiepileptics.

^b^Excluded from non-BZD antiepileptics and used to select mood stabilizers.

^c^Excluded from non-BZD anxiolytics/hypnotics/sedatives and used to select benzodiazepine derivatives in anxiolytics.

^d^Excluded from non-BZD anxiolytics/hypnotics/sedatives and used to select benzodiazepine derivatives in hypnotics/sedatives (N05CD) and benzodiazepine-related drugs (N05CF).

ATC, Anatomical Therapeutic Chemical Classification System; BZD, benzodiazepines or benzodiazepine-related drug.
