# Supplementary material for: Benzodiazepine prescribing for children, adolescents, and young adults from 2006 through 2013: A total population register-linkage study
Source: PLoS Med. 2018 Aug 7;15(8):e1002635. doi: 10.1371/journal.pmed.1002635 (PMC6080748; doi:10.1371/journal.pmed.1002635)
Supplement: S2 Table — (DOCX) [file pmed.1002635.s004.docx]

**S2 Table. ICD-10 diagnostic codes included in the study.**

| **Study covariate** | **ICD-10 code** | **ICD-10 title** |
| --- | --- | --- |
| **Psychiatric diagnoses** |  |  |
| Substance use disorders | F10 | Mental and behavioural disorders due to use of alcohol |
|  | F11 | Mental and behavioural disorders due to use of opioids |
|  | F12 | Mental and behavioural disorders due to use of cannabinoids |
|  | F13 | Mental and behavioural disorders due to use of sedatives and hypnotics |
|  | F14 | Mental and behavioural disorders due to use of cocaine |
|  | F15 | Mental and behavioural disorders due to use of other stimulants, including caffeine |
|  | F16 | Mental and behavioural disorders due to use of hallucinogens |
|  | F17 | Mental and behavioural disorders due to use of tobacco |
|  | F18 | Mental and behavioural disorders due to use of volatile solvents |
|  | F19 | Mental and behavioural disorders due to multiple drug use and use of other psychoactive substances |
| Schizophrenia, schizotypal, and delusional disorders | F20 | Schizophrenia |
|  | F21 | Schizotypal disorder |
|  | F22 | Persistent delusional disorders |
|  | F23 | Acute and transient psychotic disorders |
|  | F24 | Induced delusional disorder |
|  | F25 | Schizoaffective disorders |
|  | F28 | Other nonorganic psychotic disorders |
|  | F29 | Unspecified nonorganic psychosis |
| Bipolar disorders | F30 | Manic episode |
|  | F31 | Bipolar affective disorder |
| Depressive disorders | F32 | Depressive episode |
|  | F33 | Recurrent depressive disorder |
|  | F34 | Persistent mood [affective] disorders |
|  | F38 | Other mood [affective] disorders |
|  | F39 | Unspecified mood [affective] disorder |

**S2 Table (cont.). ICD-10 diagnostic codes included in the study.**

| **Study covariate** | **ICD-10 code** | **ICD-10 title** |
| --- | --- | --- |
| Anxiety disorders | F40 | Phobic anxiety disorders |
|  | F41 | Other anxiety disorders |
| Obsessive-compulsive disorder | F42 | Obsessive-compulsive disorder |
| Reaction to severe stress and adjustment disorders | F43 | Reaction to severe stress, and adjustment disorders |
| Dissociative, somatoform and other neurotic disorders | F44 | Dissociative [conversion] disorders |
|  | F45 | Somatoform disorders |
|  | F48 | Other neurotic disorders |
| Mental retardation | F70 | Mild mental retardation |
|  | F71 | Moderate mental retardation |
|  | F72 | Severe mental retardation |
|  | F73 | Profound mental retardation |
|  | F78 | Other mental retardation |
|  | F79 | Unspecified mental retardation |
| Autism spectrum disorders | F84 | Pervasive developmental disorders |
| ADHD / ADD | F90 | Hyperkinetic disorders |
|  | F98.8 | Other specified behavioural and emotional disorders with onset usually occurring in childhood and adolescence (Attention deficit disorder without hyperactivity) |
| Disruptive behaviour disorders | F91 | Conduct disorders |
| Emotionally unstable personality disorder | F60.3 | Emotionally unstable personality disorder |
| Dissocial personality disorder | F60.2 | Dissocial personality disorder |
| Other personality disorders | F60.0 | Paranoid personality disorder |
|  | F60.1 | Schizoid personality disorder |
|  | F60.4 | Histrionic personality disorder |
|  | F60.5 | Anankastic personality disorder |
|  | F60.6 | Anxious [avoidant] personality disorder |
|  | F60.7 | Dependent personality disorder |

**S2 Table (cont.). ICD-10 diagnostic codes included in the study.**

| **Study covariate** | **ICD-10 code** | **ICD-10 title** |
| --- | --- | --- |
|  | F60.8 | Other specific personality disorders |
|  | F60.9 | Personality disorder, unspecified |
|  | F61 | Mixed and other personality disorders |
|  | F62 | Enduring personality changes, not attributable to brain damage and disease |
|  | F63 | Habit and impulse disorders |
|  | F64 | Gender identity disorders |
|  | F65 | Disorders of sexual preference |
|  | F66 | Psychological and behavioural disorders associated with sexual development and orientation |
|  | F68 | Other disorders of adult personality and behaviour |
|  | F69 | Unspecified disorder of adult personality and behaviour |
| Nonorganic sleep disorders and insomnias | F51 | Nonorganic sleep disorders |
|  | G47.0 | Disorders of initiating and maintaining sleep [insomnias] |
| **Epilepsy** | G40 | Epilepsy |

ADD, attention deficit disorder without hyperactivity; ADHD, attention deficit hyperactivity disorders; ICD, International Classification of Disorders.
