# Supplementary material for: Benzodiazepine prescribing for children, adolescents, and young adults from 2006 through 2013: A total population register-linkage study
Source: PLoS Med. 2018 Aug 7;15(8):e1002635. doi: 10.1371/journal.pmed.1002635 (PMC6080748; doi:10.1371/journal.pmed.1002635)
Supplement: S3 Table — (DOCX) [file pmed.1002635.s005.docx]

**S3 Table. BZD prescribing patterns by average daily dosage in 117,739 study participants during the study period (2006-2013)**

| **Covariate** | **Total *n*^a^** |  | **Average daily dosage** | | | | | | | | |
| --- | --- | --- | --- | --- | --- | --- | --- | --- | --- | --- | --- |
|  |  |  | **˂0.5 DDD (reference)** |  | **≥0.5DDD to <1.5DDD** | | |  | **≥1.5DDD** | | |
|  |  |  | ***n* (%)** |  | ***n* (%)** | **Crude**  **OR (95% CI)** | **Adjusted^b^**  **OR (95% CI)** |  | ***n* (%)** | **Crude**  **OR (95% CI)** | **Adjusted^b^**  **OR (95% CI)** |
| **Whole cohort** | 117,739 |  | 98,852 (83.96) |  | 15,885 (13.49) |  |  |  | 3,002 (2.55) |  |  |
| **Sex** |  |  |  |  |  |  |  |  |  |  |  |
| Females | 67,313 |  | 57,219 (85.00) |  | 8,697 (12.92) | 1.00 | 1.00 |  | 1,397 (2.08) | 1.00 | 1.00 |
| Males | 50,426 |  | 41,633 (82.56) |  | 7,188 (14.25) | 1.14 (1.10-1.17) | 1.29 (1.25-1.34) |  | 1,605 (3.18) | 1.58 (1.47-1.70) | 1.81 (1.69-1.95) |
| **Age at first BZD dispensation** |  |  |  |  |  |  |  |  |  |  |  |
| 0-11 years | 17,500 |  | 16,850 (96.29) |  | 607 (3.47) | 1.00 | 1.00 |  | 43 (0.25) | 1.00 | 1.00 |
| 12-17 years | 15,039 |  | 12,618 (83.90) |  | 2,069 (13.76) | 4.55 (4.15-5.00) | 2.34 (2.12-2.59) |  | 352 (2.34) | 10.93 (7.96-15.02) | 5.90 (4.26-8.17) |
| 18-24 years | 85,200 |  | 69,384 (81.44) |  | 13,209 (15.50) | 5.28 (4.86-5.74) | 2.77 (2.51-3.05) |  | 2,607 (3.06) | 14.72 (10.89-19.91) | 8.90 (6.47-12.23) |
| **Any lifetime psychiatric diagnosis^c^** | 68,476 |  | 52,308 (76.39) |  | 13,381 (19.54) | 4.75 (4.54-4.97) | 3.05 (2.91-3.20) |  | 2,787 (4.07) | 11.53 (10.03-13.26) | 7.00 (6.08-8.06) |
| **Lifetime diagnosis of epilepsy^d^** | 15,191 |  | 13,213 (86.98) |  | 1,635 (10.76) | 0.74 (0.70-0.79) | 1.01 (0.94-1.07) |  | 343 (2.26) | 0.84 (0.75-0.94) | 1.45 (1.27-1.64) |
| **Concurrent dispensation of any psychotropic medication^e^** | 89,400 |  | 71,404 (79.87) |  | 15,088 (16.88) | 7.28 (6.77-7.82) | 3.99 (3.69-4.30) |  | 2,908 (3.25) | 11.89 (9.68-14.61) | 4.88 (3.96-6.02) |

^a^Total number of individuals in each row represents 100%.

^b^Adjusted for all variables in the table.

^c^Reference category is the individuals without any lifetime psychiatric diagnosis.

^d^Reference category is the individuals without lifetime epilepsy.

^e^Reference category is the individuals without any concurrent psychotropic medication, i.e., psychotropic medication dispensed within 6 months prior to or after BZD dispensation.

BZD, benzodiazepine or benzodiazepine-related drug; OR, odds ratio.
