# Supplementary material for: Benzodiazepine prescribing for children, adolescents, and young adults from 2006 through 2013: A total population register-linkage study
Source: PLoS Med. 2018 Aug 7;15(8):e1002635. doi: 10.1371/journal.pmed.1002635 (PMC6080748; doi:10.1371/journal.pmed.1002635)
Supplement: S4 Table — (DOCX) [file pmed.1002635.s006.docx]

**S4 Table. BZD prescribing patterns by “user category” in 117,739 study participants during the study period (2006-2013).**

| **Covariate** | **Total *n*^a^** |  | **User category** | | | | | | | | |
| --- | --- | --- | --- | --- | --- | --- | --- | --- | --- | --- | --- |
|  |  |  | **Occasional users (reference)** |  | **Regular users** | | |  | **Heavy users** | | |
|  |  |  | ***n* (%)** |  | ***n* (%)** | **Crude**  **OR (95% CI)** | **Adjusted^b^**  **OR (95% CI)** |  | ***n* (%)** | **Crude**  **OR (95% CI)** | **Adjusted^b^**  **OR (95% CI)** |
| **Whole cohort** | 117,739 |  | 108,558 (92.20) |  | 7,157 (6.08) |  |  |  | 2,024 (1.72) |  |  |
| **Sex** |  |  |  |  |  |  |  |  |  |  |  |
| Females | 67,313 |  | 62,097 (92.25) |  | 4,198 (6.24) | 1.00 | 1.00 |  | 1,018 (1.51) | 1.00 | 1.00 |
| Males | 50,426 |  | 46,461 (92.14) |  | 2,959 (5.87) | 0.94 (0.90-0.99) | 1.01 (0.97-1.07) |  | 1,006 (2.00) | 1.32 (1.21-1.44) | 1.46 (1.33-1.59) |
| **Age at first BZD dispensation** |  |  |  |  |  |  |  |  |  |  |  |
| 0-11 years | 17,500 |  | 17,045 (97.40) |  | 415 (2.37) | 1.00 | 1.00 |  | 40 (0.23) | 1.00 | 1.00 |
| 12-17 years | 15,039 |  | 13,709 (91.16) |  | 1,052 (7.00) | 3.15 (2.81-3.54) | 1.48 (1.31-1.69) |  | 278 (1.85) | 8.64 (6.20-12.05) | 4.27 (3.02-6.02) |
| 18-24 years | 85,200 |  | 77,804 (91.32) |  | 5,690 (6.68) | 3.00 (2.72-3.32) | 1.51 (1.33-1.71) |  | 1,706 (2.00) | 9.34 (6.83-12.79) | 5.12 (3.65-7.17) |
| **Any lifetime psychiatric diagnosis^c^** | 68,476 |  | 60,052 (87.70) |  | 6,499 (9.49) | 7.98 (7.36-8.65) | 4.99 (4.60-5.42) |  | 1,925 (2.81) | 15.71 (12.83-19.23) | 9.07 (7.40-11.12) |
| **Lifetime diagnosis of epilepsy^d^** | 15,191 |  | 13,896 (91.48) |  | 1,038 (6.83) | 1.16 (1.08-1.24) | 1.34 (1.24-1.46) |  | 257 (1.69) | 1.00 (0.87-1.13) | 1.54 (1.33-1.79) |
| **Concurrent dispensation of any psychotropic medication^e^** | 89,400 |  | 80,308 (89.83) |  | 7,081 (7.92) | 32.77 (26.13-41.10) | 15.51 (12.34-19.50) |  | 2,011 (2.25) | 54.42 (31.54-93.90) | 21.08 (12.20-36.43) |

^a^Total number of individuals in each row represents 100%.

^b^Adjusted for all variables in the table.

^c^Reference category is the individuals without any lifetime psychiatric diagnosis.

^d^Reference category is the individuals without lifetime epilepsy.

^e^Reference category is the individuals without any concurrent psychotropic medication, i.e., psychotropic medication dispensed within 6 months prior to or after BZD dispensation.

BZD, benzodiazepine or benzodiazepine-related drug; OR, odds ratio.
