# Supplementary material for: Benzodiazepine prescribing for children, adolescents, and young adults from 2006 through 2013: A total population register-linkage study
Source: PLoS Med. 2018 Aug 7;15(8):e1002635. doi: 10.1371/journal.pmed.1002635 (PMC6080748; doi:10.1371/journal.pmed.1002635)
Supplement: S5 Table — (DOCX) [file pmed.1002635.s007.docx]

**S5 Table. Characteristics of children (0-11 years), adolescents (12-17 years), and young adults (18-24 years) *without lifetime diagnosis of epilepsy* with at least one dispensed BZD prescription in 2006-2013.**

|  | **Age at first BZD dispensation (years)** | | | |
| --- | --- | --- | --- | --- |
| **Characteristics of study sample** | **0-24** | **0-11** | **12-17** | **18-24** |
| **Total *n*** | 102,548 | 9,978 | 11,135 | 81,435 |
| **Sex (%)** |  |  |  |  |
| Males | 41.36 | 56.48 | 40.30 | 39.65 |
| Females | 58.64 | 43.52 | 59.70 | 60.35 |
| **Concurrent medication (%)** |  |  |  |  |
| *Number of classes of concurrent medication* |  |  |  |  |
| No additional class | 26.35 | 85.71 | 23.81 | 19.43 |
| One additional class | 25.86 | 10.00 | 22.31 | 28.28 |
| Two additional classes | 23.14 | 2.44 | 23.27 | 25.66 |
| Three or more additional classes | 24.66 | 1.85 | 30.62 | 26.64 |
| *Class of concurrent medication^a^* |  |  |  |  |
| Any antidepressant | 55.05 | 1.22 | 53.45 | 61.86 |
| Any psychostimulant | 7.62 | 1.98 | 15.36 | 7.25 |
| Any mood stabiliser | 8.56 | 3.64 | 12.53 | 8.63 |
| Any antiepileptic (non-BZD) | 6.38 | 1.86 | 6.70 | 6.88 |
| Any antipsychotic | 15.62 | 1.29 | 21.76 | 16.53 |
| Any anxiolytic/hypnotic/sedative (non-BZD) | 41.76 | 3.79 | 51.02 | 45.15 |
| Any analgesic | 14.99 | 6.01 | 15.20 | 16.07 |
| Any opioid | 16.18 | 1.59 | 14.36 | 18.22 |
| Any drug used in addictive disorders | 2.69 | 0.09 | 2.22 | 3.07 |

^a^Not mutually exclusive.

BZD, benzodiazepines or benzodiazepine-related drug.
