# Supplementary material for: Benzodiazepine prescribing for children, adolescents, and young adults from 2006 through 2013: A total population register-linkage study
Source: PLoS Med. 2018 Aug 7;15(8):e1002635. doi: 10.1371/journal.pmed.1002635 (PMC6080748; doi:10.1371/journal.pmed.1002635)
Supplement: S6 Table — (DOCX) [file pmed.1002635.s008.docx]

**S6 Table. Psychiatric disorders diagnosed within 6 months of BZD dispensations (n= 96,498) and lifetime diagnosis (n=102,548) in children (0-11 years), adolescents (12-17 years), and young adults (18-24 years) *without lifetime diagnosis of epilepsy* with at least one dispensed BZD prescription in 2006-2013**

| **Disorder** | **Diagnosis within 6 months of BZD dispensation^a^** | | | |  | **Lifetime diagnosis^b^** | | | |
| --- | --- | --- | --- | --- | --- | --- | --- | --- | --- |
|  | **Age at first BZD dispensation (years)** | | | |  | **Age at first BZD dispensation (years)** | | | |
|  | **0-24** | **0-11** | **12-17** | **18-24** |  | **0-24** | **0-11** | **12-17** | **18-24** |
| **Total *n*** | 96,498 | 9,390 | 10,595 | 76,513 |  | 102,548 | 9,978 | 11,135 | 81,435 |
| **Any psychiatric diagnosis (%)^c^** | 46.27 | 4.71 | 56.46 | 49.95 |  | 59.14 | 9.42 | 68.41 | 63.96 |
| Substance use disorders | 10.38 | 0.02 | 10.22 | 11.68 |  | 18.40 | 0.18 | 16.77 | 20.85 |
| Schizophrenia, schizotypal, and delusional  disorders | 3.64 | 0.01 | 4.23 | 4.00 |  | 5.02 | 0.03 | 5.22 | 5.61 |
| Bipolar disorders | 4.76 | 0.14 | 7.00 | 5.01 |  | 6.78 | 0.21 | 8.51 | 7.35 |
| Depressive disorders | 22.38 | 0.21 | 29.44 | 24.12 |  | 32.56 | 0.43 | 37.55 | 35.81 |
| Anxiety disorders | 22.46 | 0.38 | 26.29 | 24.64 |  | 32.46 | 0.87 | 34.87 | 35.99 |
| Obsessive-compulsive disorder | 2.48 | 0.20 | 3.66 | 2.60 |  | 4.14 | 0.28 | 5.35 | 4.44 |
| Reaction to severe stress and adjustment  disorders | 9.18 | 0.11 | 11.64 | 9.96 |  | 15.24 | 0.24 | 16.50 | 16.91 |
| Dissociative, somatoform and other neurotic  disorders | 1.21 | 0.10 | 2.57 | 1.16 |  | 2.80 | 0.20 | 4.17 | 2.93 |
| Mental retardation | 3.34 | 0.03 | 2.70 | 3.83 |  | 5.19 | 0.04 | 3.56 | 6.05 |
| Autism spectrum disorders | 0.20 | 0.00 | 0.17 | 0.22 |  | 0.36 | 0.00 | 0.23 | 0.42 |
| ADHD / ADD | 4.07 | 0.00 | 4.37 | 4.53 |  | 5.55 | 0.00 | 5.38 | 6.26 |
| Disruptive behaviour disorders | 1.46 | 1.98 | 3.06 | 1.17 |  | 2.64 | 3.63 | 4.69 | 2.24 |
| Emotionally unstable personality disorder | 3.87 | 2.02 | 9.01 | 3.38 |  | 6.07 | 4.09 | 12.32 | 5.45 |
| Dissocial personality disorder | 7.78 | 1.51 | 14.16 | 7.67 |  | 12.62 | 3.68 | 20.70 | 12.61 |
| Other personality disorders | 0.45 | 0.23 | 2.21 | 0.23 |  | 1.47 | 0.54 | 4.13 | 1.21 |
| Nonorganic sleep disorders and insomnias | 2.77 | 0.45 | 8.19 | 2.30 |  | 4.70 | 0.94 | 10.75 | 4.33 |

Data retrieved from the National Patient Register (NPR), which comprises clinical diagnoses, coded using the International Classification of Diseases, from inpatient care (1964 onwards) and specialist outpatient services (2001 onwards), with complete (national) coverage since 1987 and 2001, respectively. The NPR does not include data on diagnoses recorded in primary care.

^a^Data on disorders extracted for study participants who were dispensed a BZD from January 1, 2006, to June 30, 2013, to allow a 6-month time window for diagnoses after dispensation.

^b^Data on disorders extracted for all study participants if diagnosed ever between 1997 and 2013.

^c^Not mutually exclusive.

ADD, attention deficit disorder without hyperactivity; ADHD, attention deficit hyperactivity disorder; BZD, benzodiazepine or benzodiazepine-related drug.
