# Supplementary material for: Benzodiazepine prescribing for children, adolescents, and young adults from 2006 through 2013: A total population register-linkage study
Source: PLoS Med. 2018 Aug 7;15(8):e1002635. doi: 10.1371/journal.pmed.1002635 (PMC6080748; doi:10.1371/journal.pmed.1002635)
Supplement: S7 Table — (DOCX) [file pmed.1002635.s009.docx]

**S7 Table. Type of BZD medication dispensed to children (0-11 years), adolescents (12-17 years), and young adults (18-24 years) *without lifetime diagnosis of epilepsy* in 2006-2013.**

| **BZD substance** | **Age at first BZD dispensation (years)** | | | | | | | |
| --- | --- | --- | --- | --- | --- | --- | --- | --- |
|  | **0-24 (n=102,548)** | | **0-11 (n=9,978)** | | **12-17 (n=11.135)** | | **18-24 (n=81,435)** | |
|  | **%** | **Relative change from 2006 to 2013^a^** | **%** | **Relative change from 2006 to 2013 ^a^** | **%** | **Relative change from 2006 to 2013 ^a^** | **%** | **Relative change from 2006 to 2013 ^a^** |
| **Any anxiolytic** | 52.82 | +2.15 | 97.39 | -0.09 | 47.74 | +2.80 | 48.05 | +0.23 |
| Diazepam | 24.54 | -13.98 | 96.39 | +0.01 | 26.54 | -8.59 | 15.46 | -21.72 |
| Oxazepam | 28.39 | +36.92 | 0.94 | -5.04 | 23.26 | +23.40 | 32.45 | +32.80 |
| Potassium clorazepate | 0.00 | ---^b^ | 0.00 | ---^b^ | 0.00 | ---^b^ | 0.00 | ---^b^ |
| Lorazepam | 0.67 | +9.43 | 0.09 | -72.73 | 1.28 | +20.97 | 0.66 | -2.22 |
| Clobazam | 0.02 | +200.00 | 0.11 | +414.29 | 0.03 |  | 0.00 | -100.00 |
| Alprazolam | 6.55 | -36.59 | 0.10 | -20.00 | 5.85 | +8.66 | 7.43 | -37.16 |
| **Any hypnotic or sedative^c^** | 65.96 | +0.73 | 2.47 | +21.81 | 70.86 | +4.91 | 73.07 | -0.52 |
| Nitrazepam | 2.29 | -22.97 | 0.50 | +12.22 | 3.04 | +2.28 | 2.41 | -34.75 |
| Flunitrazepam | 0.91 | -49.11 | 0.02 | -49.11 | 1.04 | -8.92 | 1.00 | -47.12 |
| Triazolam | 0.32 | +4.35 | 0.05 | -100.00 | 0.55 | +19.48 | 0.32 | -24.00 |
| Midazolam | 0.16 | +380.00 | 0.97 | +225.00 | 0.38 | +362.50 | 0.03 | -33.33 |
| Zopiclone | 43.80 | +33.18 | 0.60 | -32.38 | 48.96 | +17.34 | 48.39 | +30.16 |
| Zolpidem | 30.59 | -26.78 | 0.49 | -20.95 | 33.97 | -2.72 | 33.82 | -24.52 |
| Zaleplon | 1.98 | -73.26 | 0.00 | ---^b^ | 2.12 | -39.29 | 2.20 | -69.84 |
| **Any antiepileptic** |  |  |  |  |  |  |  |  |
| Clonazepam | 1.45 | -6.62 | 1.51 | +70.30 | 1.81 | -11.85 | 1.40 | -32.27 |

^a^Relative change in proportion of individuals who were dispensed a BZD substance in 2013 from that in 2006 (the value estimated in 2006 is subtracted from that in 2013, and the result is divided by the value in 2006). Relative change is reported as a percentage, with positive quantities corresponding to increases in values over time, and negative ones corresponding to decreases.

^b^ Relative change was not calculated as no individuals in this age category were dispensed a prescription for the BZD substance in question ever during 2006–2013.

^c^ Includes benzodiazepine derivatives in hypnotics/sedatives and a group of benzodiazepine-related drugs in hypnotics/sedatives also known as Z-drugs.

BZD, benzodiazepines or benzodiazepine-related drug.
