# Supplementary material for: Benzodiazepine prescribing for children, adolescents, and young adults from 2006 through 2013: A total population register-linkage study
Source: PLoS Med. 2018 Aug 7;15(8):e1002635. doi: 10.1371/journal.pmed.1002635 (PMC6080748; doi:10.1371/journal.pmed.1002635)
Supplement: S8 Table — (DOCX) [file pmed.1002635.s010.docx]

**S8 Table. Health care provider category for where the first BZD prescription was issued for children (0-11 years), adolescents (12-17 years), and young adults (18-24 years) *without lifetime diagnosis of epilepsy* in 2006-2013.**

| **Healthcare provider category (%)** | **Age at first BZD dispensation (years)** | | | |
| --- | --- | --- | --- | --- |
|  | **0-24 (n=102,548)** | **0-11 (n=9,978)** | **12-17 (n=11,135)** | **18-24 (n=81,435)** |
| Primary care | 45.35 | 10.07 | 32.89 | 51.38 |
| Specialised care other than psychiatry^a^ | 16.26 | 81.63 | 17.14 | 8.14 |
| Psychiatric care | 38.38 | 8.30 | 49.97 | 40.48 |

^a^ BZD prescriptions were most frequently initiated by paediatricians for children and adolescents and by internists for young adults.

BZD, benzodiazepines or benzodiazepine-related drug.
