# Supplementary material for: Benzodiazepine prescribing for children, adolescents, and young adults from 2006 through 2013: A total population register-linkage study
Source: PLoS Med. 2018 Aug 7;15(8):e1002635. doi: 10.1371/journal.pmed.1002635 (PMC6080748; doi:10.1371/journal.pmed.1002635)
Supplement: S9 Table — (DOCX) [file pmed.1002635.s011.docx]

**S9 Table. BZD prescribing patterns by duration of prescription in 102,548 study participants *without lifetime diagnosis of epilepsy* during the study period (2006-2013)**

| **Covariate** | **Total *n*^a^** |  | **Duration of prescription** | | | | | | | | |
| --- | --- | --- | --- | --- | --- | --- | --- | --- | --- | --- | --- |
|  |  |  | **3 months (reference)** |  | **>3 months to ≤6 months** | | |  | **>6 months** | | |
|  |  |  | ***n* (%)** |  | ***n* (%)** | **Crude**  **OR (95% CI)** | **Adjusted^b^**  **OR (95% CI)** |  | ***n* (%)** | **Crude**  **OR (95% CI)** | **Adjusted^b^**  **OR (95% CI)** |
| **Subcohort** | 102,548 |  | 57,320 (55.90) |  | 16,523 (16.11) |  |  |  | 28,705 (27.99) |  |  |
| **Sex** |  |  |  |  |  |  |  |  |  |  |  |
| Females | 60,138 |  | 33,104 (55.05) |  | 9,607 (15.97) | 1.00 | 1.00 |  | 17,427 (28.98) | 1.00 | 1.00 |
| Males | 42,410 |  | 24,216 (57.10) |  | 6,916 (16.31) | 0.98 (0.95-1.02) | 1.12 (1.08-1.16) |  | 11,278 (26.59) | 0.88 (0.86-0.91) | 1.02 (0.99-1.05) |
| **Age at first BZD dispensation** |  |  |  |  |  |  |  |  |  |  |  |
| 0-11 years | 9,978 |  | 9,265 (92.85) |  | 329 (3.30) | 1.00 | 1.00 |  | 384 (3.85) | 1.00 | 1.00 |
| 12-17 years | 11,135 |  | 6,024 (54.10) |  | 1,831 (16.44) | 8.56 (7.58-9.67) | 3.57 (3.15-4.06) |  | 3,280 (29.46) | 13.14 (11.76-14.67) | 2.62 (2.32-2.95) |
| 18-24 years | 81,435 |  | 42,031 (51.61) |  | 14,363 (17.64) | 9.62 (8.61-10.76) | 3.97 (3.53-4.46) |  | 25,041 (30.75) | 14.37 (12.96-15.94) | 2.90 (2.59-3.24) |
| **Any lifetime psychiatric diagnosis^c^** | 60,642 |  | 25,395 (41.88) |  | 11,428 (18.85) | 2.82 (2.72-2.93) | 1.71 (1.65-1.78) |  | 23,819 (39.28) | 6.13 (5.92-6.35) | 3.35 (3.23-3.48) |
| **Concurrent dispensation of any psychotropic medication^d^** | 75,526 |  | 33,858 (44.83) |  | 14,440 (19.12) | 4.80 (4.57-5.04) | 2.97 (2.81-3.13) |  | 27,228 (36.05) | 12.77 (12.09-13.50) | 6.32 (5.96-6.70) |

^a^Total number of individuals in each row represents 100%.

^b^Adjusted for all variables in the table.

^c^Reference category is the individuals without any lifetime psychiatric diagnosis.

^d^Reference category is the individuals without any concurrent psychotropic medication, i.e., psychotropic medication dispensed within 6 months prior to or after BZD dispensation.

BZD, benzodiazepines or benzodiazepine-related drug; OR, odds ratio.
