# Supplementary material for: Benzodiazepine prescribing for children, adolescents, and young adults from 2006 through 2013: A total population register-linkage study
Source: PLoS Med. 2018 Aug 7;15(8):e1002635. doi: 10.1371/journal.pmed.1002635 (PMC6080748; doi:10.1371/journal.pmed.1002635)
Supplement: S10 Table — (DOCX) [file pmed.1002635.s012.docx]

**S10 Table. BZD prescribing patterns by average daily dosage in 102,548 study participants *without lifetime diagnosis of epilepsy* during the study period (2006-2013).**

| **Covariate** | **Total *n*^a^** |  | **Average daily dosage** | | | | | | | | |
| --- | --- | --- | --- | --- | --- | --- | --- | --- | --- | --- | --- |
|  |  |  | **˂0.5 DDD (reference)** |  | **≥0.5DDD to<1.5DDD** | | |  | **≥1.5DDD** | | |
|  |  |  | ***n* (%)** |  | ***n* (%)** | **Crude**  **OR (95% CI)** | **Adjusted^b^**  **OR (95% CI)** |  | ***n* (%)** | **Crude**  **OR (95% CI)** | **Adjusted^b^**  **OR (95% CI)** |
| **Subcohort** | 102,548 |  | 85,639 (83.51) |  | 14,250 (13.90) |  |  |  | 2,659 (2.59) |  |  |
| **Sex** |  |  |  |  |  |  |  |  |  |  |  |
| Females | 60,138 |  | 50,972 (84.76) |  | 7,926 (13.18) | 1.00 | 1.00 |  | 1,240 (2.06) | 1.00 | 1.00 |
| Males | 42,410 |  | 34,667 (81.74) |  | 6,324 (14.91) | 1.17 (1.13-1.22) | 1.33 (1.28-1.38) |  | 1,419 (3.35) | 1.68 (1.56-1.82) | 1.93 (1.78-2.09) |
| **Age at first BZD dispensation** |  |  |  |  |  |  |  |  |  |  |  |
| 0-11 years | 9,978 |  | 9,895 (99.17) |  | 77 (0.77) | 1.00 | 1.00 |  | 6 (0.06) | 1.00 | 1.00 |
| 12-17 years | 11,135 |  | 9,186 (82.50) |  | 1,672 (15.02) | 23.39 (18.58-29.44) | 6.34 (5.01-8.02) |  | 277 (2.49) | 49.73 (22.14-111.70) | 9.03 (4.00-20.38) |
| 18-24 years | 81,435 |  | 66,558 (81.73) |  | 12,501 (15.35) | 24.13 (19.27-30.23) | 6.62 (5.27-8.34) |  | 2,376 (2.92) | 58.87 (26.41-131.21) | 11.02 (4.91-24.69) |
| **Any lifetime psychiatric diagnosis^c^** | 60,642 |  | 46,121 (76.05) |  | 12,045 (19.86) | 4.68 (4.46-4.91) | 2.87 (2.73-3.02) |  | 2,476 (4.08) | 11.59 (9.97-13.48) | 6.90 (5.92-8.05) |
| **Concurrent dispensation of any psychotropic medication^d^** | 75,526 |  | 59,485 (78.76) |  | 13,472 (17.84) | 7.61 (7.07-8.20) | 3.76 (3.48-4.07) |  | 2,569 (3.40) | 12.55 (10.17-15.49) | 4.88 (3.94-6.05) |

^a^Total number of individuals in each row represents 100%.

^b^Adjusted for all variables in the table.

^c^.Reference category is the individuals without any lifetime psychiatric diagnosis.

^d^.Reference category is the individuals without any concurrent psychotropic medication, i.e., psychotropic medication dispensed within 6 months prior to or after BZD dispensation.

BZD, benzodiazepines or benzodiazepine-related drug; OR, odds ratio.
