# Supplementary material for: Benzodiazepine prescribing for children, adolescents, and young adults from 2006 through 2013: A total population register-linkage study
Source: PLoS Med. 2018 Aug 7;15(8):e1002635. doi: 10.1371/journal.pmed.1002635 (PMC6080748; doi:10.1371/journal.pmed.1002635)
Supplement: S11 Table — (DOCX) [file pmed.1002635.s013.docx]

**S11 Table. BZD prescribing patterns by “user category” in 102,548 study participants *without lifetime diagnosis of epilepsy* during the study period (2006-2013).**

| **Covariate** | **Total *n*^a^** |  | **User category** | | | | | | | | |
| --- | --- | --- | --- | --- | --- | --- | --- | --- | --- | --- | --- |
|  |  |  | **Occasional users (reference)** |  | **Regular users** | | |  | **Heavy users** | | |
|  |  |  | ***n* (%)** |  | ***n* (%)** | **Crude**  **OR (95% CI)** | **Adjusted^b^**  **OR (95% CI)** |  | ***n* (%)** | **Crude**  **OR (95% CI)** | **Adjusted^b^**  **OR (95% CI)** |
| **Subcohort** | 102,548 |  | 94,662 (92.31) |  | 6,119 (5.97) |  |  |  | 1,767 (1.72) |  |  |
| **Sex** |  |  |  |  |  |  |  |  |  |  |  |
| Females | 60,138 |  | 55,538 (92.35) |  | 3,703 (6.16) | 1.00 | 1.00 |  | 897 (1.49) | 1.00 | 1.00 |
| Males | 42,410 |  | 39,124 (92.25) |  | 2,416 (5.70) | 0.93 (0.88-0.98) | 1.03 (0.97-1.08) |  | 870 (2.05) | 1.38 (1.25-1.51) | 1.53 (1.39-1.68) |
| **Age at first BZD dispensation** |  |  |  |  |  |  |  |  |  |  |  |
| 0-11 years | 9,978 |  | 9,947 (99.69) |  | 25 (0.25) | 1.00 | 1.00 |  | 6 (0.06) | 1.00 | 1.00 |
| 12-17 years | 11,135 |  | 10,137 (91.04) |  | 785 (7.05) | 30.81 (20.67-45.93) | 4.05 (2.70-6.07) |  | 213 (1.91) | 34.83 (15.47-78.45) | 4.00 (1.77-9.07) |
| 18-24 years | 81,435 |  | 74,578 (91.58) |  | 5,309 (6.52) | 28.32 (19.11-41.98) | 3.78 (2.53 (5.64) |  | 1,548 (1.90) | 34.41 (15.43-76.73) | 4.05 (1.81-9.08) |
| **Any lifetime psychiatric diagnosis^c^** | 60,642 |  | 53,332 (87.95) |  | 5,615 (9.26) | 8.63 (7.87-9.47) | 4.83 (4.40-5.30) |  | 1,695 (2.80) | 18.24 (14.41-23.10) | 10.09 (7.96-12.79) |
| **Concurrent dispensation of any psychotropic medication^d^** | 75,526 |  | 67,722 (89.67) |  | 6,049 (8.01) | 34.38 (27.15-43.53) | 14.74 (11.60-18.71) |  | 1,755 (2.32) | 58.18 (32.97-102.66) | 21.61 (12.21-38.23) |

^a^Total number of individuals in each row represents 100%.

^b^Adjusted for all variables in the table.

^c^Reference category is the individuals with no lifetime psychiatric diagnosis.

^d^Reference category is the individuals without any concurrent psychotropic medication, i.e., psychotropic medication dispensed within 6 months prior to or after BZD dispensation.

BZD, benzodiazepines or benzodiazepine-related drug; OR, odds ratio
