# Supplementary material for: Benzodiazepine prescribing for children, adolescents, and young adults from 2006 through 2013: A total population register-linkage study
Source: PLoS Med. 2018 Aug 7;15(8):e1002635. doi: 10.1371/journal.pmed.1002635 (PMC6080748; doi:10.1371/journal.pmed.1002635)
Supplement: S1 Text — (DOCX) [file pmed.1002635.s014.docx]

**S1 Text. Study protocol and analysis plan**

The following document presents a pre-specified analysis plan for the current study and includes the modifications made in response to the comments from the Editors and Reviewers.

**RATIONALE**

Widespread use of benzodiazepines and benzodiazepine-related drugs (hereafter “BZD”) has long raised public health concerns, owing to the risks of developing tolerance, dependence, withdrawal syndromes, and severe adverse effects, particularly among long-term users [1-3]. Current knowledge on BZD prescribing practices, incidence and prevalence rates, and patterns of use mainly rely on data on adults and elderly, while evidence pertaining to the corresponding issues in younger ages is limited [4]. Over the last two decades, prevalence rates of BZD use have increased in children and adolescents in various Western countries [5-7], while remaining stable or decreasing in others [6,8,9]. Noteworthy, recent European data on new BZD users reports a low and decreasing incidence in the age group below 18 years [10]. This, in light of a stable or increasing prevalence, points towards a risk for chronic BZD use in this population. It remains largely unexplored what factors influence BZD utilization and shape the different usage among young people.

**AIMS**

1. To explore the annual prevalence of BZD dispensations and secular trends among individuals aged below 25 years during 2006-2013.

2. To investigate how dispensation of BZD in children, adolescents, and young adults varies by individual user’s characteristics (gender, psychiatric morbidity and epilepsy, concurrent use of psychotropic medication), type of BZD drugs, and characteristics of the prescribers.

3. To establish the patterns of BZD dispensation with regard to duration of use and dispensed dosage and to explore associations between different patterns and characteristics of the user.

**METHODS**

**Study design**

This is a retrospective, total population register-based study.

**Data sources and data acquisition**

(1) The Swedish Prescribed Drug Register (since July 2005 onwards): data on prescribed medications dispensed across all pharmacies in Sweden, ATC Classification System codes, dosage, dispensed amount, dispensation date, and prescriber’s characteristics [11].

(2) The National Patient Register (inpatient care [1964-onwards] and specialist outpatient care [2001-onwards]): clinical diagnoses, ICD codes, and dates of diagnoses [12].

(3) The Total Population Register (from 1968 onwards): age and sex of inhabitants [13].

The register linkage is performed through the unique personal identification number assigned to all Swedish citizens and residents [14].

**BZD dispensations**

From the Swedish Prescribed Drug Register: ATC-codes for benzodiazepine derivatives in anxiolytics (N05BA), hypnotics/sedatives (N05CD), and antiepileptics (N03AE), and benzodiazepine-related drugs in hypnotics/sedatives (N05CF).

**Study participants**

From the Swedish Prescribed Drug Register: all individuals aged 0-24 years who had been dispensed BDZ at least once between January 1, 2006 and December 31, 2013.

From the Total Population Register: gender and birth year. The birth year is used to calculate age at the first and each consecutive BZD dispensations, and categorised into children (0-11 years), adolescents (12-17 years), and young adults (18-24 years). For the analyses over the whole period categorization is based on the age at the first BZD dispensation. For annual prevalence, the actual age under the specific year is used.

**MEASURES**

**BZD usage patterns**

From the Swedish Prescribed Drug Register: dates of each BZD dispensations between January 1, 2006 and December 31, 2013. For each study participant establish:

(1) an “*individual treatment period*” as the length of time between the first and the final BZD dispensation in a sequence of BZD dispensations if the gap between two consecutive dispensations did not exceed 6 months *plus* 3-month medication supply per prescription, according to the Swedish Pharmaceutical Benefits (15).

(2) an “*average daily dosage*” of BZD for each individual period by dividing the total amount of BZD dispensed in a corresponding treatment period by the length of that period.

***Modification suggested by the reviewers:*** The strategy for calculating an “individual treatment period” and an “average daily dosage” has been visualized and exemplified by the diagram (see S1 Fig for details).

Three patterns of BZD use:

(1) *Duration of BZD use,* based on the length of an individual treatment period and categorised into: 3 months (the reference), >3 to ≤6 months, or >6 months.

(2) *Used dosage,* based on an average daily dosage calculated for the treatment period and categorized into: <0.5 DDD (the reference), ≥0.5 to <1.5 DDD, or ≥1.5 DDD.

(3) *User category,* reflects the benchmarks introduced by the Swedish National Board of Health and Welfare (16) and combines the dosage and duration of BZD use, and categorized into: “regular user” (≥0.5 to <1.5 DDD consumed for ≥1 year), “heavy user” (≥1.5 DDD consumed for ≥1 year), or otherwise, “occasional user” (the reference). If multiple treatment periods are identified for the study participant, the highest category of each usage pattern is used for analysis.

***Modification suggested by the reviewers:*** The strategy for defining the patterns of BZD use and for selecting the highest category of each usage pattern for the analysis has been visualized and exemplified by the diagram (see S1 Fig for details).

**Concurrent psychotropic medication**

From the Swedish Prescribed Drug Register: dates of each psychotropic drug dispensations between July 1, 2005 and June 30, 2014 to cover 6-month period before and after each BZD dispensation. Psychotropic drugs include: antidepressants [N06A]; psychostimulants (centrally acting sympathomimetic) [N06BA], mood stabilisers [N03AF01, N03AF02, N03AG01, N03AX09, N03AN01], non-BZD antiepileptics [N03 (except N03AE) and (except N03AF01, N03AF02, N03AG01, N03AX09, N03AN01)], antipsychotics [N05A], non-BZD anxiolytics/hypnotics/sedatives [N05B (except N05BA), N05C (except N05CD, N05CF)], analgesics [N02B, N02C], opioids [N02A], and drugs used in addictive disorders [N07B].

**Psychiatric diagnoses and epilepsy**

From the National Patient Register: ICD-10 codes and dates for psychiatric diagnoses and epilepsy from January 1, 1997 to December 31, 2013 (for “lifetime diagnoses”) and from January 1, 2006 to June 30, 2013 (for “diagnosed 6 months prior to or after BZD dispensation”). Diagnoses include: substance use disorders [F10-F19], schizophrenia, schizotypal, and delusional disorders [F20-F29], bipolar disorders [F30, F31], depressive disorders [F32-F39], anxiety disorders [F40, F41], obsessive-compulsive disorder [F42], reaction to severe stress and adjustment disorders [F43], dissociative, somatoform and other neurotic disorders [F44, F45, F48], mental retardation [F70-F73, F78, F79], autism spectrum disorders [F84], ADHD / ADD [F90, F98.8], disruptive behaviour disorders [F91], dissocial personality disorder [F60.2], emotionally unstable personality disorder [F60.3], other personality disorders [F60.0, F60.1, F60.4-F60.9, F61-F66, F68, F69], nonorganic sleep disorders and insomnias [F51, G47.0], epilepsy [G40].

**Health care provider category**

From the Swedish Prescribed Drug Register: specialty of the prescriber who issued the first BZD dispensation categorised as primary care, specialized care other than psychiatry, and psychiatric care.

**STATISTICAL ANALYSIS**

(1) Descriptive analysis:

(a) annual prevalence: a proportion of individuals who were dispensed BZD at least once during each year from 2006 to 2013 to the total number of inhabitants of the same age in Sweden in the corresponding year. Denominator is retrieved from Statistics Sweden (17). Multiple BZD dispensations within the same years are counted only once. Calculated separately for children, adolescents, and young adults.

(b) attributes of prevalent BZD use: a proportion of a certain attribute of BZD use (gender, concurrent use of psychotropic drugs, lifetime and “within 6 months” psychiatric diagnoses and epilepsy, type of BZD drugs, and prescriber’s characteristics) per 100 study participants. Calculated within each year (the denominator includes individuals with at least one BZD dispensation in a given year) and across the whole period (the denominator includes all individuals with at least one BZD dispensation between 2006-2013).

(c) relative change in annual prevalence or in proportions of attributes of BZD use from 2013 to those in 2006 (the referent value): the value estimated in 2006 is subtracted from that in 2013 and the result is divided by the value in 2006.

***Modification suggested by reviewers:*** the original calculation of 95% confidence intervals for annual prevalence, test for linearity and chi-squared test for independence have been omitted.

(2) Multinomial regression:

***Modification suggested by reviewers:*** The original age- and gender-adjusted model was replaced by the crude and multivariate models (as below).

(a) univariate model: odds ratio and 95% confidence intervals for crude associations between each of the user pattern *and* age at the first BZD dispensation, gender, lifetime psychiatric diagnoses and epilepsy, and concurrent use of psychotropic drugs.

(b) multivariate model: variables are included if found significant in the univariate models, or/and if they fulfilled the criteria for being confounders (18).

**Sensitivity analysis**

A series of analyses are conducted by repeating the main analyses in a sub-population restricted to individuals without lifetime diagnoses of epilepsy to capture the BZD use for indications other than seizures.

**REFERENCES**

1. Dell'osso B, Lader M. Do benzodiazepines still deserve a major role in the treatment of psychiatric disorders? A critical reappraisal. Eur Psychiatry. 2013;28(1):7-20.

2. Kurko TA, Saastamoinen LK, Tahkapaa S, Tuulio-Henriksson A, Taiminen T, Tiihonen J, et al. Long-term use of benzodiazepines: Definitions, prevalence and usage patterns - a systematic review of register-based studies. Eur Psychiatry. 2015;30(8):1037-47.

3. Lader M. Benzodiazepines revisited--will we ever learn? Addiction. 2011;106(12):2086-109.

4. Witek MW, Rojas V, Alonso C, Minami H, Silva RR. Review of benzodiazepine use in children and adolescents. Psychiatr Q. 2005;76(3):283-96.

5. Alessi-Severini S, Bolton JM, Enns MW, Dahl M, Collins DM, Chateau D, et al. Use of benzodiazepines and related drugs in Manitoba: a population-based study. CMAJ Open. 2014;2(4):E208-16.

6. Huerta C, Abbing-Karahagopian V, Requena G, Oliva B, Alvarez Y, Gardarsdottir H, et al. Exposure to benzodiazepines (anxiolytics, hypnotics and related drugs) in seven European electronic healthcare databases: a cross-national descriptive study from the PROTECT-EU Project. Pharmacoepidemiol Drug Saf. 2016;25 Suppl 1:56-65.

7. Hugtenburg JG, Heerdink ER, Egberts AC. Increased psychotropic drug consumption by children in the Netherlands during 1995-2001 is caused by increased use of methylphenidate by boys. Eur J Clin Pharmacol. 2004;60(5):377-9.

8. Steffenak AK, Wilde-Larsson B, Nordstrom G, Skurtveit S, Hartz I. Increase in psychotropic drug use between 2006 and 2010 among adolescents in Norway: a nationwide prescription database study. Clin Epidemiol. 2012;4:225-31.

9. O'Sullivan K, Reulbach U, Boland F, Motterlini N, Kelly D, Bennett K, et al. Benzodiazepine prescribing in children under 15 years of age receiving free medical care on the General Medical Services scheme in Ireland. BMJ Open. 2015;5(6):e007070.

10. Benard-Laribiere A, Noize P, Pambrun E, Bazin F, Verdoux H, Tournier M, et al. Trends in incident use of benzodiazepines and Z-drugs in France from 2006 to 2012: a population-based study. Pharmacoepidemiol Drug Saf. 2017;26(2):162-9.

11. Wettermark B, Hammar N, Fored CM, Leimanis A, Otterblad Olausson P, Bergman U, et al. The new Swedish Prescribed Drug Register--opportunities for pharmacoepidemiological research and experience from the first six months. Pharmacoepidemiol Drug Saf. 2007;16(7):726-35.

12. Ludvigsson JF, Andersson E, Ekbom A, Feychting M, Kim JL, Reuterwall C, et al. External review and validation of the Swedish national inpatient register. BMC Public Health. 2011;11:450.

13. Ludvigsson JF, Almqvist C, Bonamy AK, Ljung R, Michaelsson K, Neovius M, et al. Registers of the Swedish total population and their use in medical research. Eur J Epidemiol. 2016;31(2):125-36.

14. Ludvigsson JF, Otterblad-Olausson P, Pettersson BU, Ekbom A. The Swedish personal identity number: possibilities and pitfalls in healthcare and medical research. Eur J Epidemiol. 2009;24(11):659-67.

15. The Dental and Pharmaceutical Benefits Agency. Ordinance (2002:687) on Pharmaceutical Benefits, etc. 2002 [Available from: <http://www.tlv.se/Upload/English/ENG-ordinance-2002-687.pdf>.

16. Quality and efficiency in Swedish health care. Regional comparisons 2010. Swedish Association of Local Authorities and Regions and the National Board of Health and Welfare 2011;219-236. Available from: <http://www.socialstyrelsen.se/lists/artikelkatalog/attachments/18336/2011-5-18.pdf>.

17. Statistics Sweden. Statistical database. Population statistics. Population by age and sex. Year 1860-2016. 2017. Available from: <http://www.statistikdatabasen.scb.se/pxweb/sv/ssd/START__BE__BE0101__BE0101A/BefolkningR1860/table/tableViewLayout1/?rxid=6e7ce440-36a7-4ef6-9d3f-84749100819e>.

18. Greenland S, Lash TL. Bias analysis. In: Rothman KJ, Greenland S, Lash TL, editors. Modern Epidemiology. Philadelphia: Lippincott Williams and Wilkins; 2008. pp.345-80.
